# Integrated microfluidic approach for quantitative high-throughput measurements of transcription factor binding affinities

**DOI:** 10.1093/nar/gkv1327

**Published:** 2015-12-03

**Authors:** Yair Glick, Yaron Orenstein, Dana Chen, Dorit Avrahami, Tsaffrir Zor, Ron Shamir, Doron Gerber

**Affiliations:** 1 Mina and Evrard Goodman life science faculty, Bar Ilan University, Ramat-Gan, 5290002, Israel; 2 Blavatnik School of Computer Science, Tel-Aviv University, Tel-Aviv, 69978, Israel; 3 Department of Biochemistry & Molecular Biology, Life Sciences Institute, Tel-Aviv University, Tel-Aviv, 69978, Israel

## Abstract

Protein binding to DNA is a fundamental process in gene regulation. Methodologies such as ChIP-Seq and mapping of DNase I hypersensitive sites provide global information on this regulation *in vivo*. *In vitro* methodologies provide valuable complementary information on protein–DNA specificities. However, current methods still do not measure absolute binding affinities. There is a real need for large-scale quantitative protein–DNA affinity measurements. We developed QPID, a microfluidic application for measuring protein–DNA affinities. A single run is equivalent to 4096 gel-shift experiments. Using QPID, we characterized the different affinities of ATF1, c-Jun, c-Fos and AP-1 to the CRE consensus motif and CRE half-site in two different genomic sequences on a single device. We discovered that binding of ATF1, but not of AP-1, to the CRE half-site is highly affected by its genomic context. This effect was highly correlated with ATF1 ChIP-seq and PBM experiments. Next, we characterized the affinities of ATF1 and ATF3 to 128 genomic CRE and CRE half-site sequences. Our affinity measurements explained that *in vivo* binding differences between ATF1 and ATF3 to CRE and CRE half-sites are partially mediated by differences in the minor groove width. We believe that QPID would become a central tool for quantitative characterization of biophysical aspects affecting protein–DNA binding.

## INTRODUCTION

Protein–DNA interaction is a fundamental process in the living cell. Many proteins interact with DNA to modulate and affect a wide variety of cellular processes including DNA replication, repair and recombination. The expression of genes requires transcription by RNA polymerase. The transcription process is regulated by a variety of associated proteins, referred to generally as transcription factors (TFs). Transcription factors are found in all living organisms and their number increases with genome size. In fact, larger genomes tend to have higher fraction of TFs among their genes. Approximately 10% of genes in the human genome encode for TFs, which makes them the largest family in the proteome (1,2). In humans, it is estimated that 200–300 transcription factors bind core promoter elements and are considered components of the general transcriptional machinery (3). In addition, there are about 1,400 transcription factors with sequence-specific DNA-binding preferences that regulate only a subset of genes by binding to site-specific *cis*-elements (3). Interestingly, the site-specific factors tend to be expressed either in all (or most) tissues or in one or two tissues, suggesting either a very broad or very specific function (4).

Our understanding of the interactions between transcriptional regulators and their targets is still insufficient. Current methodologies for characterization of TF binding sites (BSs) suffer from low resolution, low throughput and limited dynamic range (5–7). As a result, weaker regulatory interactions other than those occurring at high-affinity BSs are largely ignored and are not well understood (8). Moreover, recent evidence suggests that knowledge of both strongly and weakly bound sequences and their interaction affinities is required for an accurate understanding of transcriptional regulation (8–10) and may allow closely related TFs to mediate different transcriptional responses (11). In addition, quantitative models require both strongly and weakly bound sequences and their binding affinities to recapitulate transcriptional responses (12–16).

Current methods for studying protein–DNA interactions and for characterization of TFBSs can be divided into two main groups: *in vivo* methods such as Chromatin immunoprecipitation (ChIP) based methods (17–19), DNAse footprinting (20) and *in vitro* quantitative methods, such as electrophoretic mobility shift assays (EMSA) (21) and Surface Plasmon Resonance (SPR) (22,23). *In vivo* and *in vitro* methods complement each other. However, the above-mentioned *in vitro* methods lack the required throughput needed for answering genome-wide questions and inferring accurate binding models. The throughput issue was addressed by development of several methods including one-hybrid systems (24,25), high-throughput systematic evolution of ligands by exponential enrichment (HT-SELEX) (26–28), cognate site identifier (CSI) (29), protein binding microarrays (PBMs) (30–32), genomic-context PBMs (gcPBMs) (33,34) and microfluidic-based microarray systems (10). For example, Afek et al. recently demonstrated using gcPBMs (7) that nonspecific DNA sequences possessing certain repeat symmetries, when present outside of specific TFBSs, statistically control TF−DNA binding preferences. While these methods measure protein DNA-binding specificity well, they still lack quantitative affinity measurements.

EMSA is the gold standard protocol for determining the binding potential of a DNA sequence to a protein (35–37). This method is considered a qualitative assay although under appropriate conditions it can provide quantitative data for determining binding stoichiometries, affinities and kinetics (35). Efforts to make EMSA more quantitative include combining mass spectrometry and dried EMSA gels (38), using multiplexed competitor (39) and improving the separation efficiency by using Capillary Electrophoresis (CE) (40,41). However, the throughput of these methods is still low.

Combining EMSA with microfluidics increased both the quantitative capacity and throughput by shortening the runtime from 1h to 30s (42) and increasing analytical throughput (43,44). For example, a 384-plex radial microfluidic capillary electrophoresis tool (45) or 768 wells gel array fabricated in the PAG sheet (45). Recently, Pan *et al*. (46) introduced the fsPAG-EMSAs, a photo-patterned free-standing polyacrylamide gel array that acts as a chassis for 96 concurrent EMSAs. These combined EMSA approaches increase throughput and precision, but still depend on separation efficiency of PAGE. Sample loading and protein requirements become limiting factors at this point. Effectively, throughput is only increased by about one order of magnitude, which is still not suitable for large–scale screening assays. Recently, Geertz et al. developed the k-MITOMI platform that measures kinetic data (47). However, the highly detailed understanding of the kinetics comes at the expense of throughput.

In this study, we present a high throughput microfluidic platform for Quantitative Protein Interaction with DNA (QPID). QPID is an integrated microfluidic-based assay that can perform up to 4096 parallel measurements on a single microfluidic device and quantitatively calculate the affinity of TFs to a variety of DNA elements.

QPID fills the gap between quantitative high-throughput specificity measurements (PBMs, gcPBM etc.) and small-scale detailed kinetics technologies (k-MITOMI). To date, affinities can only be measured in low-throughput, and while PBM binding intensities correlate with *K*_d_ data (30), they are not direct affinity measurements. Here we demonstrated two QPID applications.

First, we characterized the binding of four cAMP response element (CRE) TF complexes to 32 oligonucleotides at 32 different concentrations in a single experiment. QPID produced measurements of four protein complexes against 32 different DNA sequences, each in 32 different concentrations within a single device. The CRE consists of an eight base-pair palindrome: TGACGTCA (48–50), yet, in many cases the site consists of only half of the consensus CRE site, CGTCA. The half-sites are potential TFBSs, albeit with lower affinity (50). c-Jun binds as a homodimer to the AP-1 element (TGAGTCA) as well as to CRE, while c-Fos fails to dimerize and displays no apparent affinity for either the AP-1 element or the CRE. AP-1 comprises of a complex between c-Jun and c-Fos (51), which binds both CRE (52,53) and AP-1 BSs with high affinity (54). We found that binding of ATF1, but not of AP-1, to CRE half-site is highly affected by the genomic context, in concordance with PBM and ChIP-seq experiments.

In the second application, we characterized the binding of ATF1 and ATF3 to different genomic CRE and CRE half-sites elements. Here measurements of one protein against 128 different DNA sequences, each in 32 different concentrations, were performed within a single device. We found that differences in affinity levels of ATF1 and ATF3 to the same genomic binding sites explain their different *in vivo* binding. These differences can be accurately modeled using DNA shape features.

## MATERIALS AND METHODS

### Microfluidic device fabrication

The two-layer device was designed in AutoCAD software (Autodesk, Inc.) and each layer reproduced as a chrome mask at 40000 dpi (Fineline-Imaging). Flow and control molds were fabricated on 4″ silicon wafers using positive (SPR 220–7.0) and negative (SU-8) photoresists, respectively. The microfluidic devices were fabricated on silicone molds as previously described (10,55–56). Briefly, each device consists of two aligned PDMS layers, the flow and the control layer. PDMS (60g) at ratio of 5:1 was cast on the control mold and degassed. Inlet holes were then punched. PDMS (21g) at ratio of 20:1 was spin-coated on the flow mold. Both molds were semi-cured at 80°C for 30 min. The control PDMS patterns were de-molded and inlets were punched for the control layer. Control and flow layers were then assembled and cured at 80°C for 2h (56).

### CRE elements mini-library preparation

CRE elements in their genomic-context promoter sequence (Mus musculus, Chromosome 1, NC_000067.6 (131019845..131024970), Chromosome 17, NC_000083.6 (35199367..35202007), Chromosome 6, NC_000072.6 (52313498..52318389)) were synthesized (IDT), hybridized to a Cy5-labeled primer and extended using Klenow fragment (exo-) (New England Biolabs) to produce Cy5-labeled dsDNA (10) (Table 1). Cy5-labeled dsDNA oligonucleotides were diluted to a final concentration of 2 μM and a serial of 32 dilutions ranging from 2 μM down to 0.0156 μM were prepared. Each sample contained 0.125% Poly ethylene glycol (Peg, Sigma-Aldrich) and 1.25 mg/ml D-trehalose dihydrate (Sigma-Aldrich) in dH2O preventing irreversible binding of the DNA to the printed slide as well as for visualization during alignment of the device to the DNA array. A negative control sample with no DNA was included. The oligonucleotides were spotted onto epoxy coated glass substrates (CEL Associates) with a MicroGrid 610 (Bio Robotics) microarrayer using SMT-S75 silicone pins (Parallel Synthesis, USA). Column and row pitch corresponded to the specific device. The microfluidic device that was used contains 64 columns and 64 rows with a pitch of 280 μm by 560 μm, respectively.

**Table 1.** DNA sequences used for the QPID array

oligo #	oligosequnece
1	GGCCACTACCGCTTCCTCCACATGACGTCATGGTTTTCTCCACCAAGGAAGT
2	TTATGACCTGGGAGTGACGTCATGGAATCCACAGA
3	GGCCACTACCGCTTCCTCCACATGAGTCATGGTTTTCTCCACCAAGGAAGT
4	TTATGACCTGGGAGTGAGTCAATGGAATCCACAGA
5	GGCCACTACCGCTTCCTCCACATGGCGTCATGGTTTTCTCCACCAAGGAAGT
6	TTATCCACTTGCGCTCGCCGAGTGGCGTCACCAGCGGTACTGTAATGACGAT
7	GGCCACTACCGCTTCCTCCACAAATAAAATTGGTTTTCTCCACCAAGGAAGT
8	GGCCACTACCGCTTCCTCCACATGAGATCATGGTTTTCTCCACCAAGGAAGT
9	GGCCACTACCGCTTCCTCCACATGTCTACATGGTTTTCTCCACCAAGGAAGT
10	GCAGGGACCCAAAGCAGCAGCCTGAGCTCATGATCAGAGTGAAAGGAGAAGG
11	CAGGGACCCAAAGCAGCAGCCTGTCTACATGATCAGAGTGAAAGGAGAAGGc
12	TTGGCCCCAGATTGCCACAGAATCCTGGTGGGGACGACGGGGGAGAGATTCC
13	CCACGTCATTATGACCTGGGAGTGCGTGAATGGAATCCACAGATGAGGGCCc
14	CCAAAAATTTATGACCTGGGAGTGCGTGAATGGAATCCACAGATGAGGGCCc
15	TTATGACCTGGGAGTGCGTGAATGGAATCCACAGA
16	TTATGACCTGGGAGTAAATGAATGGAATCCACAGA
17	TTATGACCTGGGAGAATAAAATTGGAATCCACAGA
18	AGCCCATTTATCCACGTCATTATGACCTGGGAG
19	AGCCCATTTATCCAAAAATTTATGACCTGGGAG
20	GTAATGCAGAAGTTCATTCCGACCAGTTCTTTAGCGCTTACAATGCAAAAA
21	GTAATGCAGAAAAAATTTCCGACCAGTTCTTTAGCGCTTACAATGCAAAAA
22	GTAATGCAGAAGTTCATAATAAATGTTCTTTAGCGCTTACAATGCAAAAAc
23	GTAATGCAGAAGTTCATTCCGACCAGTTCTTTAATAAATCAATGCAAAAAc
24	AAAAAAAAAAAGAAAGAAATTAAACTCAAAAATTGCATGGTTTAGAAGAGGG
25	AAAAAAAAAAAGAAAGAAATTAAAAAATAAAATTGCATGGTTTAGAAGAGGG
26	AAGCGGAAAGACAGAGTCACCACTACGTCACGTGGAGTCCGCTTTACAGACT
27	AAGCGGAAAGACAGAGTCACCAAATAAAATCGTGGAGTCCGCTTTACAGACT
28	GTGTGCGTGCTCTGAGCAGCGAGCACGTCAGACTGCGCCCAGTGGGGAGAGG
29	GTGTGCGTGCTCTGAGCAGCGAAATAAAATGACTGCGCCCAGTGGGGAGAGG
30	CACATGAGATCATGGGAATTTCCACCAAGGAAGTTTTCCGAGGGTTGAATGAGA
31	CACATGAGATCATAGATTTCGAAACCAAGGAAGTTTTCCGAGGGTTGAATGAGA
32	CTCCGGCGGTATGAC

### Transcription factor ‘synthetic genes’ assembly

N terminal cMyc and C terminal 6*HIS or N terminal HA and C terminal V5 TFs ‘synthetic genes’ were created by using a two-step assembly polymerase chain reaction (PCR) approach as described in Glick *et al*. 2012 (56). Briefly, in the first PCR step, two epitope tags were added to each gene, C-myc in the N-terminus and His in the C-terminus, or V5 in the N-terminus and HA in the C-terminus. The first PCR products served as templates for the second PCR, in which we added the 5′ UTR (T7 promoter) and 3′ UTR (T7 terminator) for each gene. The PCR products were filtered in multi-well 10k filter plates (AcroPrep™, PALL) and eluted with 40 μl DDW.

### *In vitro* protein expression

TFs containing 3′-HIS&5′-cMyc were expressed in a tube using rabbit reticulocyte quick coupled transcription and translation reaction (TNT, Promega). The expression was performed in a final volume of 12.5 μl including 1 μg of DNA. The tube was incubated at 32°C for 2.5 h with agitation (600 rpm). To form heterodimers, a second TF with 3′-HA&5′-V5 was expressed in tube and incubated with the first TF, for dimerization, at 32°C with agitation (600 rpm) for 1 h.

### Surface chemistry

To derivatize the slide surface, biotinylated-BSA (1 μg/μl, Thermo) was flown through the device for 30 min allowing the binding of the BSA to the epoxy surface. On top of the biotinylated-BSA, 0.5μg/μl of Neutravidin (Pierce) was added for 30 min. The ‘button’ valve, a micromechanical valve used for both the surface chemistry and MITOMI (55), was then closed and biotinylated-BSA was flown over for 30 min passivating the rest of the device. Following passivation, the ‘button’ valve was released and a flow of 0.2 μg/μl biotinylated anti-HIS antibody (Qiagen) was applied. The antibody bound specifically to the exposed Avidin surface under the ‘button’ creating an anti-HIS tag array. Hepes (50 mM, Biological Industries) was used for washing unreacted substrates after each of the different surface chemistry steps.

### Protein DNA interaction assay

In each experiment, ≈25 μl of extract (≈50 ng of protein) was loaded into the device. Introduction of 3′-HIS&5′-cMyc or 3′-HA&5′-V5 TFs complex into the DNA chambers solubilize spotted DNA, allowing TFs and DNA to interact. TF–DNA complexes were then captured on the chip surface beneath the ‘button’ valve during a 1 hour incubation period. Next, MITOMI was performed by closing the ‘button’ valve to trap the interactions. We then washed out protein complexes and DNA, not trapped by MITOMI. TFs were labeled with anti-c-Myc-Cy3 (Sigma) or anti-HA Alexa 488 (Cell Signaling) antibodies, which bound the corresponding epitope on the respective TFs. Proteins expression levels and interacting DNA signals were measured with a microarray scanner (LS Reloaded, Tecan) using a 488 nm laser and 535 filter, 532 nm laser and 575 nm filter or 633 nm laser and 695 nm filter. By using fluorescent labeled antibody and Cy-5 labeled probes we can quantify the affinity. Cy3\Alexa 488 intensities under the ‘button’ valve reflect the number of surface-bound protein molecules; Cy5 intensities under the ‘button’ valve reflect the number of DNA molecules bound by surface-immobilized protein. Therefore, the ratio of Cy5 to Cy3 fluorescence is proportional to the number of DNA molecules bound per protein, namely, protein fractional occupancy. Cy5 intensities within the DNA chamber reflect the amount of soluble DNA available for binding.

### Printed DNA concentration

To determine actual on-chip printed DNA concentration, Cy5 labeled oligonucleotides with known concentrations (0.005–1 μM) were introduced into the device. Cy5 intensity was measured with a microarray scanner (LS Reloaded, Tecan) using 633 nm laser and 695 nm filter and a calibration curve was plotted. The concentration of spotted DNA in each chamber was then calculated according to this standard curve.

### Imaging & data analysis

Scanner images were analyzed using GenePix7.0 software (Molecular Devices) as previously described (10). Briefly, two different images were analyzed: Alexa 488/Cy3 emission image was used to determine protein expression levels, while Cy5 emission image was used to determine interacted DNA. The data from both images were extracted and the interaction ratios between the DNA and protein signals under the ‘button’ valve were calculated. Protein–DNA interactions affinity (*K*_d_) was determined by fitting results using non-linear least squares minimization (http://statpages.org/nonlin.html).

### Comparing QPID ATF1 binding preferences to PBM and ChIP measurements

QPID measurements of ATF1 binding to two oligonucleotides containing the CRE half-sites (oligonucleotides 5 and 6, Figure 3) were compared to available *in vivo* and *in vitro* experiments based on local DNA shape features. Such features have been widely used in recent years to model protein DNA-binding preferences based on data from high-throughput experiments (57). The shape features were computed using DNAshape (58) and compared to shapes features obtained from PBM and ChIP binding. The features are helix twist, minor groove width, roll and propeller twist (HelT, MGW, Roll and ProT, respectively). Each of these four features is assigned a real value for each position within a predetermined distance k base-pairs from the core BS. Hence, all the shape features are summarized as a 4(2k+w)-long vector for a w-long core. For the CRE half-site, we used PBM local DNA shape features already computed in TFBSshape (33). These were based on 1647 BSs identified in the PBM experiment Atf1_3026.3_v1_deBruijn.txt (11). Normalized Euclidean distances (i.e. divided by the square root of the vector length) between the feature vector of each oligonucleotide and the average PBM-based feature vector were computed. A similar analysis was performed for the ChIP-seq experiment wgEncodeAwgTfbsSydhK562Atf106325UniPk.narrowPeak downloaded from ENCODE (59). Peaks that contained the full site TGACGTCA were filtered out, and all other peaks were aligned by the half-site CGTCA (considering both orientations). This included 4310 BSs. Flanks of 10 bp on each side were used for the ChIP-seq analysis. The same analysis was performed on PBM experiment Atf1_3026.3_v2_deBruijn.txt (1619 BSs) and ChIP-seq experiment, accession number ENCSR000DNZ (7545 BSs) (Supplementary Figure S1 and Table S1).

### ATF1 and ATF3 *in vivo* binding library design

We used ChIP-seq and DNase-seq experiments to identify *in vivo* BSs of ATF1 and ATF3 as well as accessible unbound sites. ChIP-seq experiments on K562 cells were downloaded from ENCODE (59). The peaks of each experiment were the bound sites (accession numbers ENCFF002CVM and ENCFF002CVN for ATF1 and ATF3, respectively). DNase-seq experiments on K562 cells were downloaded from ENCODE as well (accession number ENCFF001UWN). We used peaks that contain one of the binding sites, CRE or CRE half-site, in either orientation. We extracted 200 bp centered around the first occurrence of the BS (CRE or CRE-hs). For CRE-hs, we excluded sequences that had a CRE full-site in the 15 bp flanks. Unbound sites were defined as sites that were extracted from the DNase-seq experiment, but were not found in the ChIP-seq experiments. See Supplementary Table S2 for the complete set of sequences and Supplementary Table S3 for the library used in the experiment.

### DNA shape features model inference

For ATF1 and ATF3 binding affinities to CRE half-site BSs, a model based on DNA shape features was inferred. DNA shape features are highly suitable for our case for two reasons (12). First, they are a compact representation of complex sequence features in the flanking regions. Second, they provide insights into the DNA-binding mechanism of the protein. For the 107 CRE half-sites, DNA shape features were computed using DNAshape (58). Affinities were 1/*K*_d_ values, and models were inferred for ATF1, ATF3 and ATF1-ATF3 affinities. The shape features used were helix twist, minor groove width, roll and propeller twist from positions 11–16 and 19–25, inclusive, comprising the 5 bp flanks of each CRE half-site. Model inference was performed using multiple linear regression.

## RESULTS

### QPID overview and design

QPID is a platform for measuring the binding energy landscapes of transcription factors, including protein complexes. To this end, we employed an integrated microfluidic platform that enables liquid manipulation in very small volumes and high throughput. The device design is based on previous work (10,55,60). For QPID, we created an array of 64 by 64 chambers, sectioned into four independent quarters. This allows the introduction and surface immobilization of up to four different proteins or complexes, one in each independent section. We also incorporated MITOMI (55,61) in the platform to increase the dynamic range and allow detection of low affinity and transient interactions by measuring binding at equilibrium. The principle behind QPID is to use co-immunoprecipitation to measure DNA binding to an immobilized protein at different DNA concentrations. For each of the 4096 experiments, we directly measure the DNA concentration in solution and the fraction bound to protein (DNA/Protein Ratio), using fluorescent labeling.

**Figure 1.**
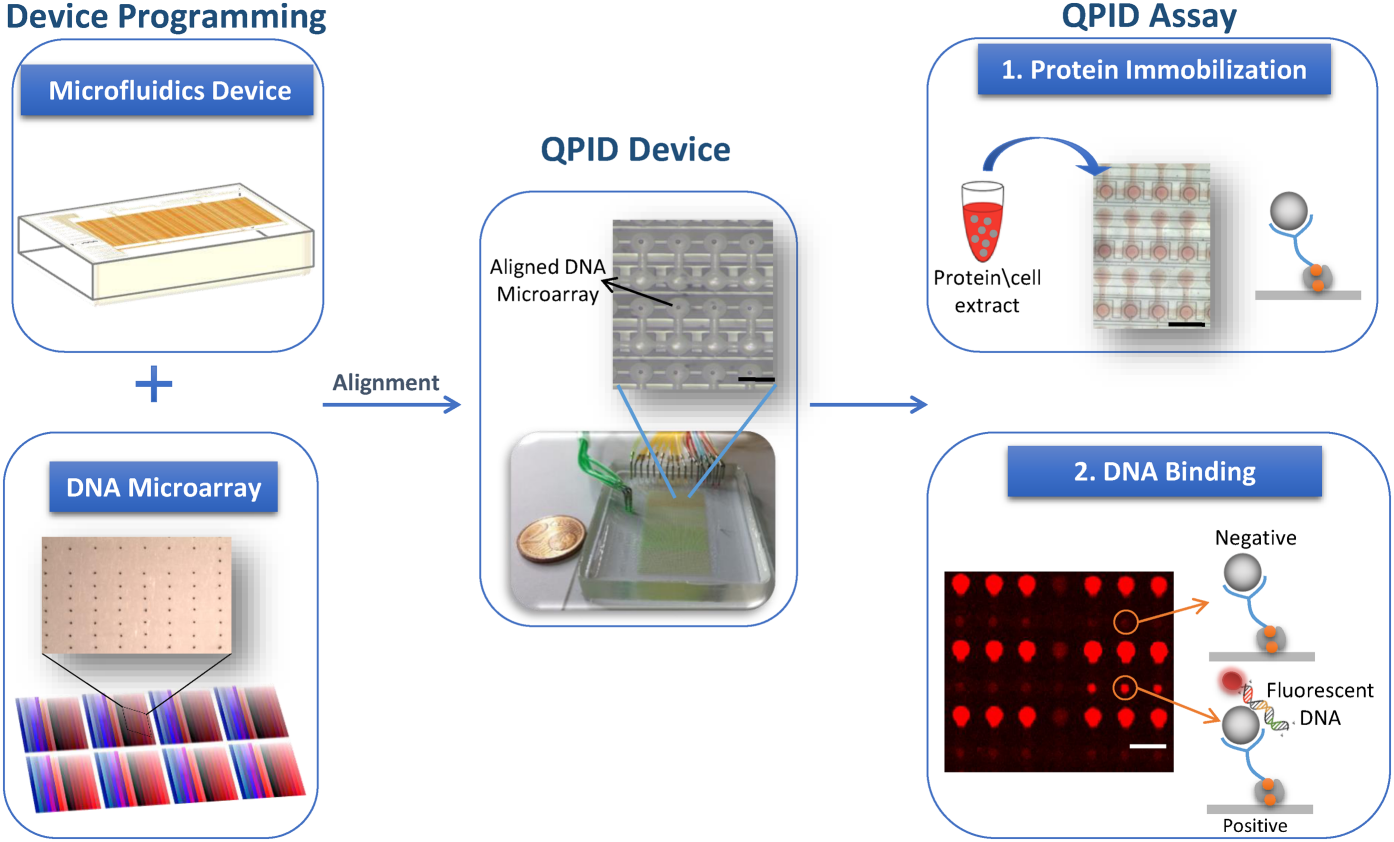
QPID system overview. QPID experiments are programmed by aligning and bonding an integrated microfluidic device containing thousands of micromechanical valves (upper left) with a DNA microarray (lower left). Each color in the microarray has a gradient and represents a different DNA sequence at different concentrations. A typical QPID Device (middle) containing 32 different oligonucleotides at 32 different concentrations in four independent identical blocks is ready for performing 4096 individual quantitative binding experiments. Zoom in on a device layout shows the DNA microarray locked within the microfluidic chambers (upper middle). 1. We express the TFs in tubes using *in vitro* transcription and translation. We load the proteins or cell extracts (with over expressed proteins) onto the QPID device and immobilize them to the surface (upper right). 2. In the DNA binding assay (bottom right), the fluorescent DNA oligonucleotides are incubated with the TFs, MITOMI is performed and fluorescent images are taken. We measure the affinity of the TFs to each of the oligonucleotides at equilibrium and calculate the dissociation constant.

We made sample loading compatible with high throughput by microarraying the DNA samples in advance. We programmed the microfluidic device (Figure 1) with 32 different probes, in 32 different concentrations, with 4 repeats each. All together results from a single QPID device are comparable to 4096 EMSA assays. The proteins are loaded into the device from designated inputs during the experiment. The modularity of our platform allows screening the four DNA repeats against one TF or each repeat against a different TF. We can express the TF in a tube using an *in vitro* transcription and translation systems. Alternatively, we can use our microfluidic device as a purification column and enrich the TF or protein complex by directly immobilizing them from cell extracts.

**Figure 2.**
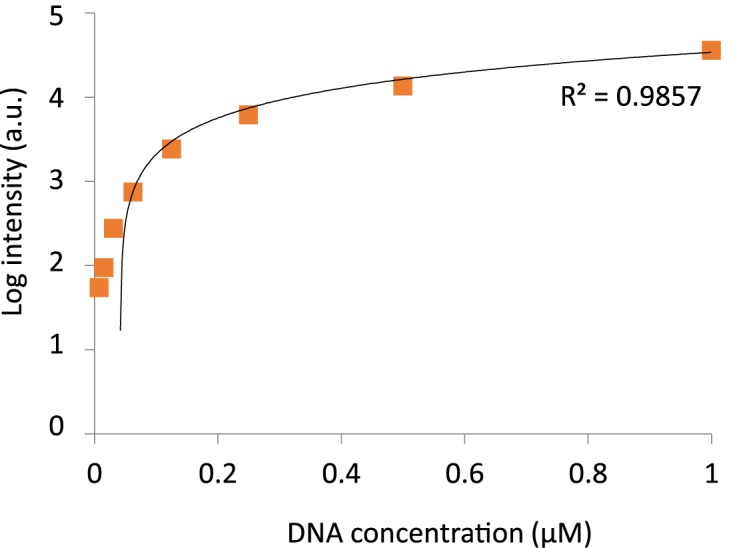
Calibration curve for on-chip DNA concentrations. Cy5 labeled oligonucleotides at concentrations ranging between 0.005–1 μM were introduced into the device. Cy5 intensity was measured and a calibration curve (*y* = 35579x - 1471) was plotted (*n* = 128).

We created a standard calibration curve to determine DNA concentrations on chip. To this end, we loaded Cy5 labeled oligonucleotides with known concentrations (0.005–1 μM) into an empty microfluidic device. Cy5 intensity was measured for each of the concentrations and a calibration curve was plotted (Figure 2). Using this standard curve, we calculated the actual concentration of soluble DNA in each QPID DNA chamber. Protein–DNA interaction affinities (*K*_d_) were calculated by fitting the DNA–protein binding results to a binding model, using non-linear least squares minimization (10).

### QPID protein–DNA interaction measurements

To evaluate the performance of QPID, we chose to use two related proteins families as models for hetero- and homo- dimer complexes. The proteins are cAMP-responsive nuclear factor 1 (ATF1) and c-Jun and c-Fos, both members of the AP-1 family. We measured their binding energy landscapes to a set of oligonucleotides based on variations of the CRE and CRE half-site.

**Figure 3.**
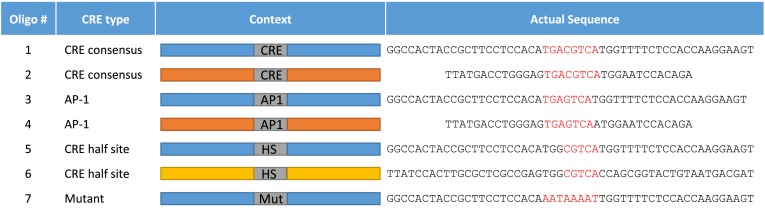
CRE elements sequences. CRE elements were tested for binding in the context of two different DNA scaffolds. The elements tested included CRE wild type, AP-1 wild type, CRE half-site and a mutated CRE. The DNA scaffolds were taken from the genomic loci of Mus musculus, Chromosome 1, NC_000067.6 (131019845..131024970) (blue), Mus musculus Chromosome 17, NC_000083.6 (35199367..35202007) (Orange), Chromosome 6, NC_000072.6 (52313498..52318389) (yellow).

**Figure 4.**
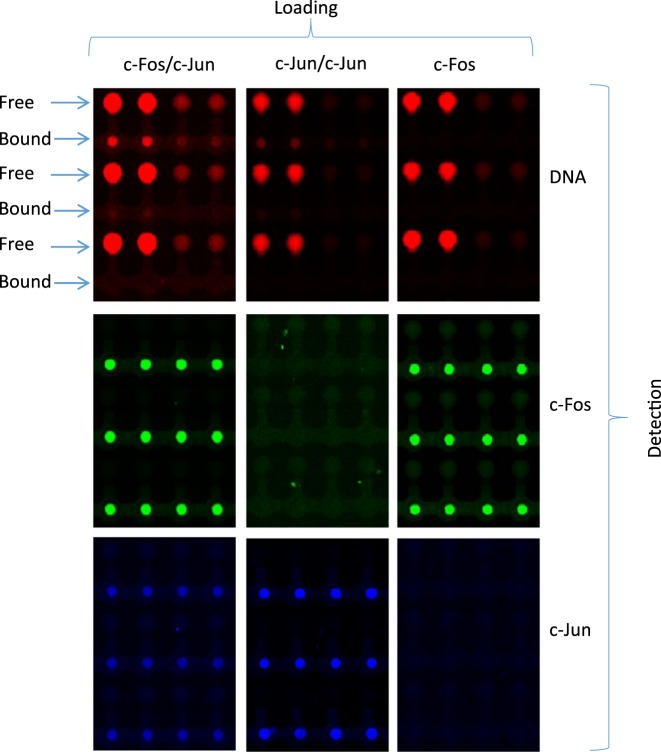
Fluorescent image of protein–DNA interaction (upper), c-Fos expression (middle) and c-Jun expression (lower). Each row is a different DNA sequence at 4 different concentrations. Each column is a different TF. c-Jun/c-Fos heterodimers (AP-1 complex) (left) interacted with DNA in high affinity, while c-Jun/c-Jun homodimers (middle) interacted in low affinity and c-Fos monomer (right) did not interact with DNA.

We synthesized a library of Cy5-labeled dsDNA probes that cover different CREs under different promoter contexts (see Materials and Methods, Table 1). The library included the CRE 8 bp consensus, CRE half-site and AP-1 consensus BS (Figure 3). We programmed QPID with a dilution series for each oligonucleotide. The surface under each ‘button’ valve was derivatized with anti-HIS antibodies (introduced to the chip from outside). Then, the *in vitro* expressed and tagged TFs - ATF1, c-Jun, c-Fos and AP-1 complex (c-Jun\c-Fos heterodimer), were applied onto the device and immobilized on the anti-HIS surfaces. We immobilized each TF in a different quarter of the device. After incubation with the DNA oligonucleotides, MITOMI was performed (55) and the quantity of trapped molecules under each ‘button’ was measured. Free DNA concentration was subsequently quantified from each DNA chamber. We labeled c-Fos with c-Myc Cy3 antibody and c-Jun with V5 FITC antibody. Raw images for specific cases of the binding assay demonstrate QPID capabilities (Figure 4). By using three different colors, we were able to distinguish between each component of a heterodimer binding to DNA (c-Jun/c-Fos). Each panel represents a binding experiment against three different DNA sequences at different concentrations. Within each panel, we see concentration dependent binding and DNA sequence specificity. We can also see examples of strong, medium and no binding for the heterodimer, homodimer and monomer, respectively. There was literally no observable background for the homodimer or monomer and no cross contamination between adjacent chambers. We observed a very high signal to noise ratio, about 100 to 1 for the protein signals under each ‘button’ valve and their local background.

**Figure 5.**
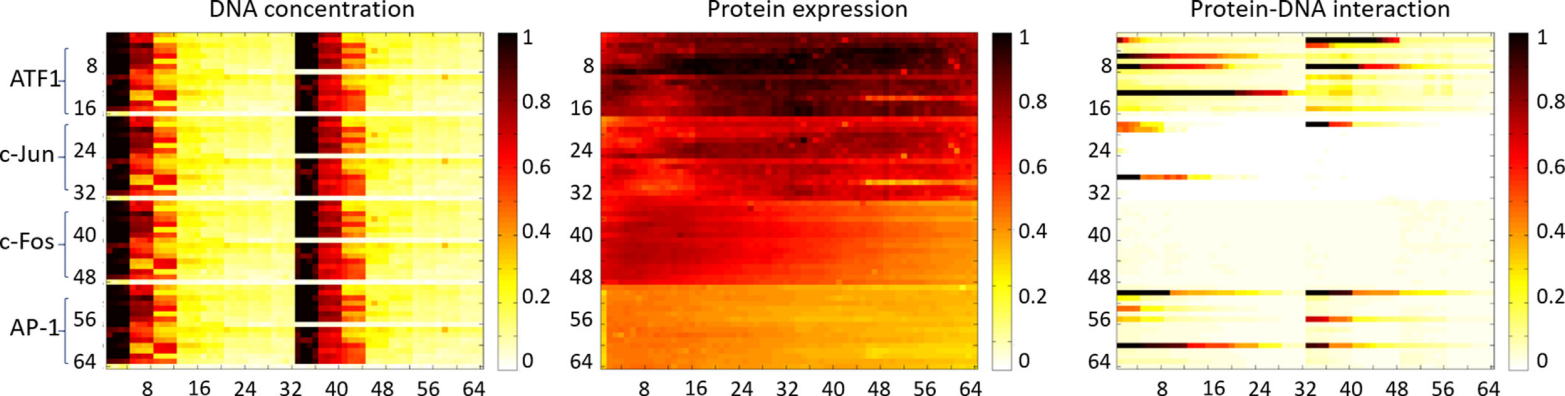
Heat maps of QPID analysis. Maps are shown for ATF1, c-Jun, c-Fos and AP-1 TF complexes, ordered top to bottom. DNA concentration (μM) in solution for the various oligonucleotides is shown on the left panel. Protein signals are shown in the middle panel (fluorescent intensity, arbitrary units). DNA binding to the protein complexes is shown in the right panel.

**Figure 6.**
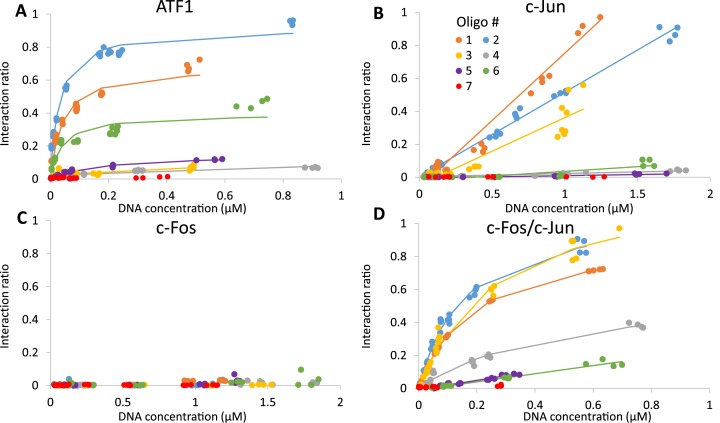
Quantitative analysis *K*_d_'s. Affinity measurements between ATF1 **(A)**, c-Jun **(B)**, c-Fos **(C)**, AP-1 **(D)** and CRE sequences. We programmed the QPID device with different CRE consensus sequences. Each TF was immobilized to the device surface, flooding DNA chambers solubilized spotted DNA, allowing TF and DNA to interact. Protein expression levels (Cy3) and interacting DNA signals (Cy5) were measured. Interaction ratio – Cy5\Cy3 (colors refer to oligonucleotide number in Figure 3).

Figure 5 shows heat maps summarizing a QPID experiment. They show, for each of the 4096 cells, DNA concentrations in solution, protein expression level and intensity of protein–DNA interactions. QPID directly measures the concentration of free DNA in solution for each of the 4096 experiments. Immobilization resulted in a relatively uniform protein distribution, with standard deviation of 6–40% of the mean. To account for this variability, we divided the concentration of bound DNA by the expression level of the protein in each cell. In Figure 6, we present the binding curves and non-linear least square fitting for each TF with each of the oligonucleotides in Figure 3. Dissociation constants (*K*_d_) ranged between concentrations of 0.03–50 μM (Table 2). The limit for detection was in accordance with Maerkl et al. (55).

**Table 2.** *K*_d_ values (μM) for binding of ATF1, c-Jun, c-Fos and AP-1 TFs to different CRE consensus sequences

Oligo #	BS	ATF1	c-Jun	c-Fos	c-Fos/c-Jun
1	CRE	0.04	1.3	-	0.19
2	CRE	0.03	2.4	-	0.15
3	AP-1	11	2.5	-	0.27
4	AP-1	17	-	-	0.50
5	HS	0.17	-	-	3.5
6	HS	0.04	-	-	4.0
7	Mutant	-	-	-	-

Results were fitted using nonlinear least square minimization. QPID's sensitivity is limited to 50 μM for quantitative affinity measurement. Weaker interactions were marked as ‘-’.

We found that ATF1 has high affinity to the CRE (0.035 ± 0.005 μM) and to the half-site CRE (0.105 ± 0.065 μM) while its affinity to the AP-1 consensus element was lower by two orders of magnitude (14 ± 3 μM) (Figure 6A). c-Jun homodimers had low affinity to the CRE and AP-1 element (2 ± 0.6μM) and we observed no binding to the CRE half-site (Figure 6B). No binding was detected for the c-Fos homodimer (Figure 6C) while the c-Jun\c-Fos heterodimer showed high affinity (0.27 ± 0.13 μM) to the CRE and AP-1 elements but low affinity (3.75 ± 0.25 μM) to the CRE half-site (Figure 6D). The *K*_d_ values are summarized in Table 2.

**Figure 7.**
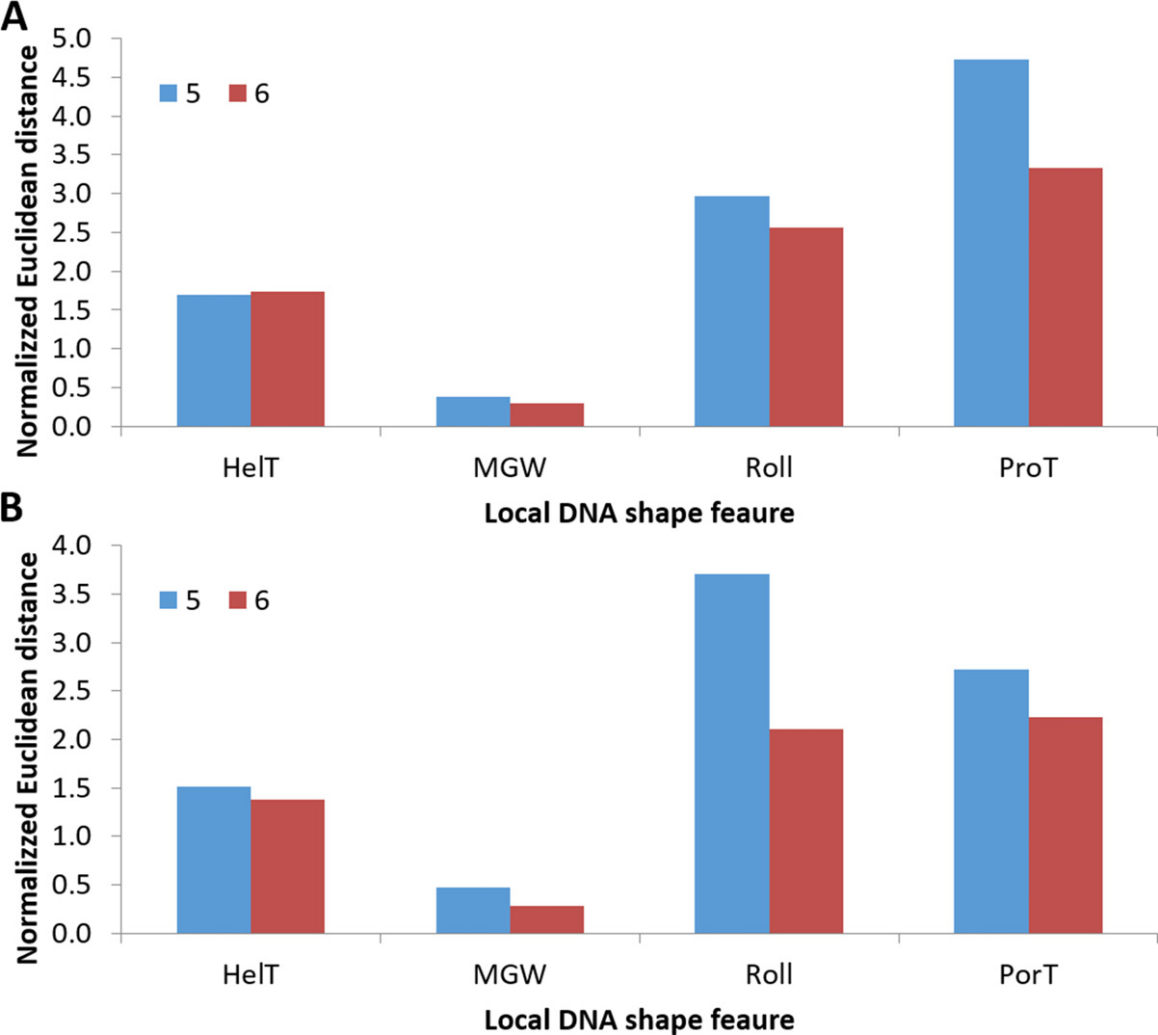
Similarity of QPID ATF1 binding preferences to PBM and ChIP-seq. Normalized Euclidean distance between DNA local shape feature predictions of oligonucleotide 5 and 6 and average shape feature predictions of CRE half-sites binding sites (TGACG) were calculated (see Materials and Methods). **(A)** Distance to an average of 1647 BSs measured by PBM. **(B)** Distance to an average of 4310 BSs measured by ChIP-seq. HelT, MGW, Roll and ProT: helix twist, minor groove width, roll and propeller twist.

Interestingly, we found that TF binding affinities to the same core consensus vary significantly depending on its genomic context. This includes the binding of ATF1 to oligonucleotide 5 and 6, both containing the CRE half-site element. This phenomenon has been observed previously in other high-throughput *in vitro* methods, such as PBM, HT-SELEX and gcPBM (34,62–63). To validate our observation, we compared available *in vitro* and *in vivo* data with the binding affinities of ATF1 to oligonucleotides 5 and 6 (see Materials and Methods). We used local DNA shape features as they were shown to accurately model the binding preferences in the sequence regions flanking the core BS (26,34,64). Our results show that the difference in binding affinities between oligonucleotides 5 and 6 is in concordance with both *in vivo* and *in vitro* experiments (Figure 7, Supplementary Figure S1 and Table S1). The distance of the feature vector of oligonucleotide 5 from the vector obtained using ChIP-seq and PBM measurements of the site was significantly greater than that of oligonucleotide 6 (*P*-value = 0.035, binomial test). We conclude that QPID accurately measures the binding affinities to the same core sequence in different genomic contexts.

### ATF1 and ATF3 *in vivo* binding differs significantly

**Figure 8.**
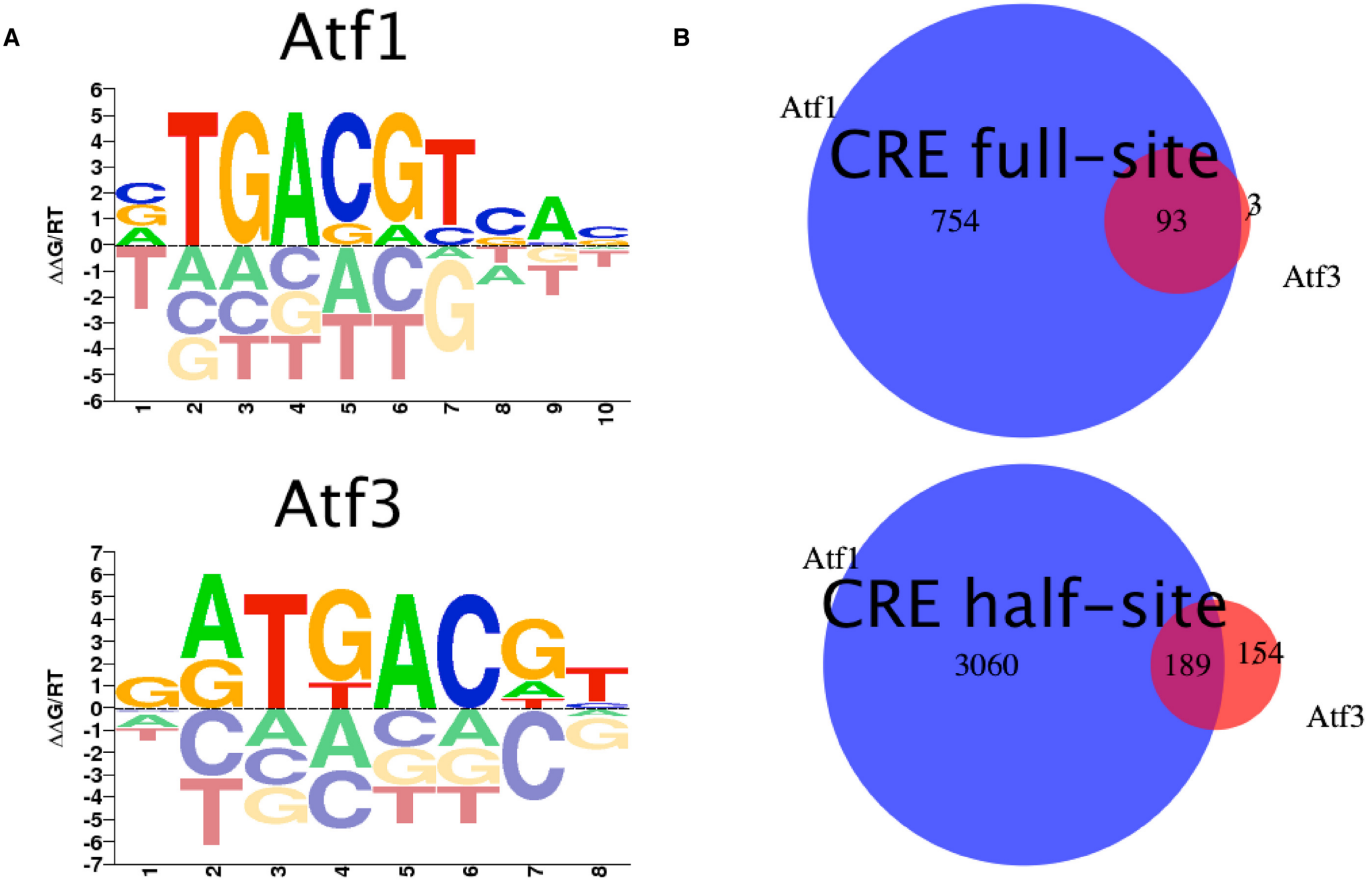
Differences in Atf1 and Atf3 *in vivo* binding. **(A)** Logo plots of public PBM-derived PWMs from CIS-BP. **(B)** Venn diagrams of Atf1 and Atf3 *in vivo* binding sites. For each binding site, CRE full- (TGACGTAC) and half-site (CGTAC), the peaks containing it were extracted from a ChIP-seq experiment. Number of unique and shared binding sites are reported.

One of the factors affecting *in vivo* binding is protein competition. Proteins in the same family prefer to bind the same high-affinity sites *in vitro*, but show different occupancy *in vivo*. It is unclear what determines protein occupancy of competing proteins. To answer this question for two proteins of the human bZIP TF family, we analyzed the binding of ATF1 and ATF3. Both TFs bind the canonical CRE element TGACGTCA, and may also bind the CRE half-site, albeit with lower affinity. Since these proteins have very similar binding preferences as represented by their PWMs (Figure 8A), their *in vivo* binding to genomic sequences is expected to be very similar.

Despite the similar binding preferences of ATF1 and ATF3, their *in vivo* BSs only partially overlap. We analyzed ChIP-seq data from K562 cell line, filtered to chromatin-accessible sites. We focused on the CRE full- and half-sites, separately. For the CRE full site 847 sites bound by ATF1, compared to 96 by ATF3, but only three of the latter are bound solely by ATF3. 575 accessible sites contained the CRE full-site but were neither bound by ATF1 nor ATF3. For the CRE half-site 3249 sites were bound by ATF1 while only 343 by ATF3, and of those 154 were bound by both. 10 251 accessible sites were unbound by both proteins (Figure 8B and Supplementary Table S2).

### ATF1 and ATF3 *in vivo* binding differences explained by QPID

We created a library to measure the binding affinities of ATF1 and ATF3 proteins to genomic sequences. The library was designed to cover both CRE full and half-sites in different genomic contexts. We selected 14 CRE full-sites: 3 bound solely by ATF1, 3 bound solely by ATF3, 3 bound by both and 5 unbound. We selected 107 CRE half-sites: 34 bound by ATF1, 34 bound by ATF3, 34 bound by both and 5 unbound. Additional 5 sequences that did not include any of the sites were used as controls. Complete sequences are in Supplementary Table S3. We programmed QPID with a dilution series for each oligonucleotide. The surface under each ‘button’ valve was derivatized as previously described and *in vitro* expressed and tagged ATF1 or ATF3 were applied onto the device. After incubation with the DNA oligonucleotides, MITOMI was performed (55), the quantity of trapped molecules under each ‘button’ was measured and free DNA concentration was subsequently quantified from each DNA chamber (Supplementary Figure S2). Dissociation constants (*K*_d_) for each TF with each of the oligonucleotides were calculated using non-linear least square fitting (Supplementary Figures S3 and S4). Complete results of binding affinities are in Supplementary Table S4.

**Figure 9.**
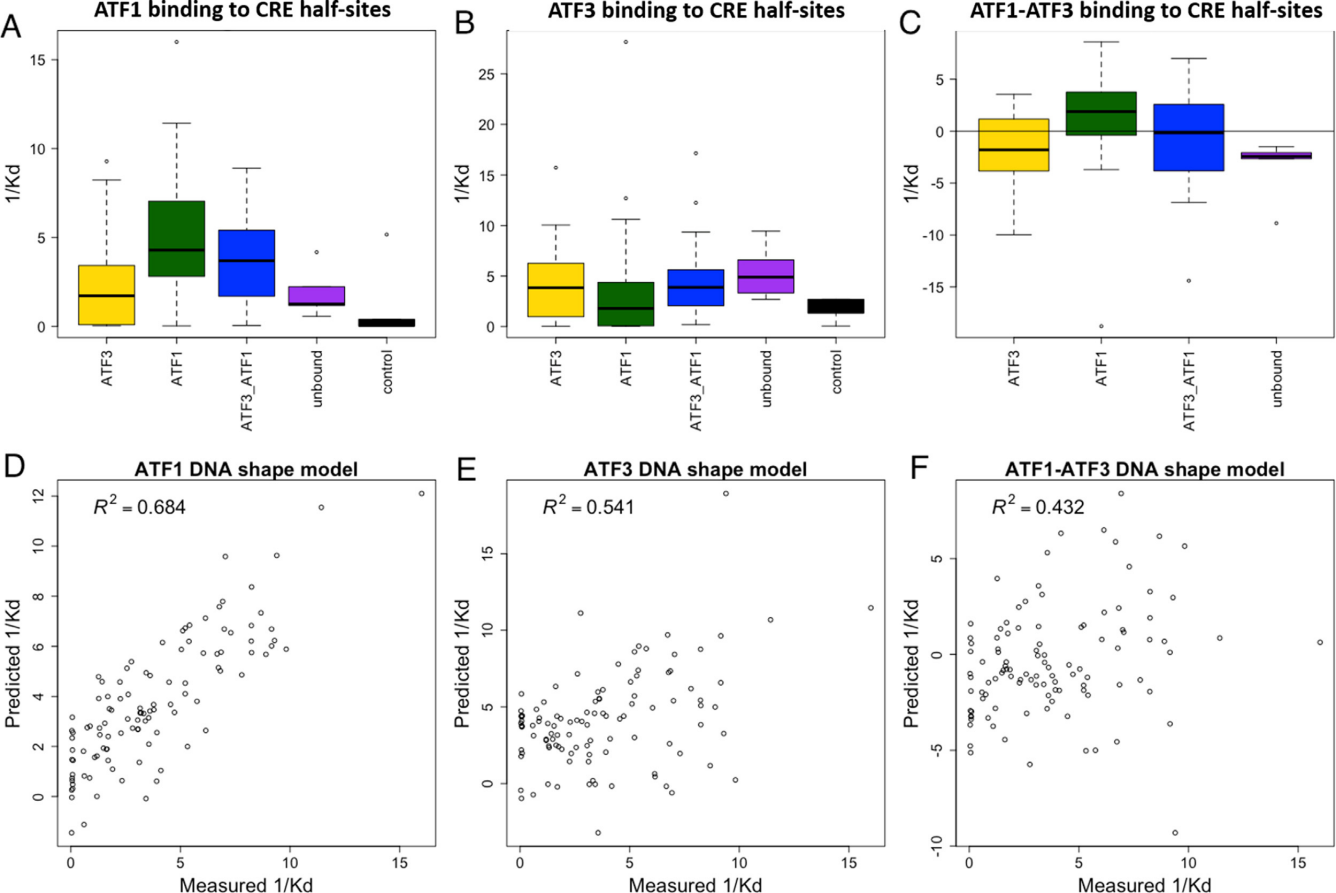
QPID measurements explain *in vivo* binding differences between ATF1 and ATF3 to CRE half-sites. **(A** and **B)** Boxplots of affinity constants (1/*K*_d_) in different binding site categories. ATF1 and ATF3 have higher affinities to sites they bound uniquely than sites bound by the other protein, unbound and controls. **(C)** Boxplots of affinity constant (1/*K*_d_) differences between ATF1 and ATF3 in different binding sites categories. ATF1 has higher affinity in sites it binds uniquely than in sites bound by ATF3. **(D** and **E)** Fit of DNA shape features- based model to measured affinities. For each protein, multiple linear regression was used to infer a binding model based on DNA shape features. **(F)** Fit of DNA shape features-based model to difference in measured affinities. Multiple linear regression was used to infer a model for the difference in binding affinities.

We first analyzed the distribution of *K*_d_ values in the different categories (Figure 9A and B for CRE half-sites and Supplementary Figure S5 for CRE). Clearly, ATF1 has higher affinity to sites bound solely by ATF1 *in vivo* than sites bound solely by ATF3 (*P*-value = 0.0003, Wilcoxon-signed rank-test). Similar results are observed for ATF3 affinities to *in vivo* ATF3-bound sites compared to ATF1-bound sites, although they were not significant (*P*-value = 0.08, Wilcoxon-signed rank-test). For sites bound by both, the affinities of each protein are somewhat dispersed between the ATF1- and ATF3-bound categories. This shows that QPID can accurately measure protein binding specificities. It also demonstrates the significant effect of flanking sequence of the core motif on binding affinities.

On top of that, we calculated the difference in affinity constants (1/*K*_d_) between the proteins for each oligo separately (Figure 9C). In this case, for ATF1-bound sites the difference in ATF1 to ATF3 affinities is the highest, in concordance with the fact that they are bound by ATF1-bound sites only. On the other hand, for ATF3-bound sites the difference is smaller, explaining their binding by ATF3 (*P*-value = 0.0001 comparing ATF1- to ATF3-bound sites, Wilcoxon-signed rank-test). We conclude that differences in binding affinities are a factor in TF binding of competing proteins. Note that this analysis can only be accomplished for absolute affinities (e.g. *K*_d_ values), as opposed to binding intensities, as they are comparable across proteins.

To try and explain the mechanism behind the binding preferences of ATF1 and ATF3 we inferred models based on DNA shape features. For the positions in the 5 bp flanking the core site we predicted four DNA shape features and inferred a model using QPID measured affinities and multiple linear regression (see Materials and Methods). In concordance with what is known about bZIP protein binding (65), the minor groove width in position 4 of the CRE-hs was the most important feature, with a preference for a narrower minor groove width. Together, the shape features were able to explain the data quite well (Figure 9D and E). Moreover, we learned a model to fit the difference in binding affinities (Figure 9F). For this model, the minor groove width was also selected to be the most important feature, hinting that the different preference for that shape feature is may be the cause for their different affinities. See Supplementary Table S5 for complete feature weights.

## DISCUSSION

DNA-binding proteins play crucial roles in many major cellular processes. Observing these binding events and measuring their affinities is key to understanding their role and function in the living cell. We have developed QPID for quantitative measurements of protein–DNA interactions. To demonstrate QPID, we used this platform to characterize the affinity of several TFs to a library of DNA elements. Overall, on each device, we performed 4096 individual experiments that covered 4 protein complexes against 32 oligonucleotides at 32 different concentrations for each of these oligonucleotides.

We found that ATF1 demonstrated high affinity to the CRE consensus element and low affinity to the CRE half-site. This is in agreement with results published by Montminy *et al*. (50), who reported that ATF1 binds both to the CRE consensus and CRE half-site, albeit with lower affinity. Interestingly, ATF1 affinity to the CRE half-site was different under the different DNA scaffolds. This observation is in concordance with available *in vivo* and *in vitro* experiments. We conclude that ATF1 binding to the CRE half-site highly depends on the promoter context as well as the CRE element sequence itself.

AP-1 complex comprises of c-Jun and c-Fos, which bind both the CRE and the AP-1 elements. c-Jun can form both hetero- and homodimers, while c-Fos can only form heterodimers. Indeed, we observed no binding for c-Fos, while c-Jun homodimers bind similarly, in low affinity, to both the CRE and AP-1 elements. This is in agreement with reports by Curran *et al*. (61). The affinity of the AP-1 complex (c-Jun/c-Fos) to the AP-1 and CRE elements was 10 times stronger, which is in agreement with a previous study (54). We also found that AP-1 binds the CRE half-site in low affinity, while c-Jun or c-Fos homodimers did not bind at all. These binding preferences have been previously reported by Foulds *et al*. (62). Overall, this is the first time an integrated microfluidic approach was used to quantify cis-regulatory site affinity and preference in high throughput.

Moreover, we were able to explain the difference in *in vivo* binding of ATF1 and ATF3 to genomic CRE and CRE half-site BSs. In our measurements, ATF1 binds with higher affinity sites that are bound solely by ATF1 compared to those that are bound by ATF3. Similarly, ATF3 binds with higher affinity sites bound solely by ATF3. On top of that, we calculated the difference in affinities for each BS, and observed that for ATF1 bound sites the difference in affinities between ATF1 and ATF3 was the highest. DNA shape-based models can partially explain the different affinities, ranking minor groove width of the BS as the most important feature in both affinity and affinity-difference models. While specificities can be measured with higher throughput in gcPBM, affinities cannot. We see the advantage in QPID in measuring absolute affinities. These allow comparison of binding preferences of competing proteins on the same scale, as we demonstrated for ATF1 and ATF3.

Our microfluidics platform, which combines a DNA microarray integrated microfluidics and an immunoprecipitation assay has several advantages over current methods. Microarraying DNA rather than using 384 microplate form bypasses the resolution problem discussed by Gaunt *et al*. (66) and increases the device density by several orders of magnitudes. This enables performing thousands of experiments in parallel on a single device. Moreover, microarraying DNA eliminates the loading time, which is a significant throughput limitation for others methods (46).

PBMs and gcPBMs are also microarray-based and can perform thousands of parallel experiments. However, they have several inherent limitations. The ‘open environment’ of the microarray chip may create cross contaminations. It also limits the sensitivity since weak interactions or proteins with fast off-rates are washed off. The need for purified proteins limits the number of proteins that are compatible with these methods. QPID overcomes the cross contamination, sensitivity and purification limitations as discussed below. Most importantly, QPID measures absolute affinities, which allow comparison between proteins that bind similar binding sites.

The usage of cell free protein systems allowed us to measure the binding energy landscapes of single TFs as well as study different homo- and hetero-dimer complexes. At the same time, it eliminates the need for protein purification. On the other hand, we are not limited to *in vitro* expressed proteins. We can apply cell extracts to the device and use the microfluidic device to purify and immobilize a target TF or complex. The ability of the microfluidics to concentrate proteins many folds enables the study of TFs that are normally in very low concentrations (67–69). This can provide us unique insight of cell processes that cannot be easily probed by current methods.

The combination between integrated microfluidics and microarraying makes QPID very flexible. We can use the same device to screen several TFs against a small set of DNA oligonucleotides or one protein against a large DNA library. The protein or proteins meet the DNA in all 4096 parallel experiments at the same time. Thus, QPID eliminates the time shift caused by pipetting each of the samples, after incubation, separately, as in other methods. Another major advantage provided by the integrated microfluidics is the use of MITOMI (55). MITOMI allows the molecular trapping of interactions and thus a snapshot of the interactions at equilibrium. This is very different from other methods, in which washing steps significantly reduce the sensitivity to low affinity interactions or to interactions with fast off rates. The latter are almost always missed by conventional high throughput methods such as gcPBMs (7,33–34). QPID's sensitivity ranges from strong 10^−9^ molar interactions to very weak 5 × 10^−5^ molar.

Replacement of the gold standard gel shift assay, which is at the base of many other methods (42–46,66), with immunoprecipitation and MITOMI (55) bypasses several performance issues. For example, dependence on sample electrical properties, the interplay between gel structure geometry and running buffer recipe, and the time dependent electrical properties of the system. In addition, our gel-free approach allows significant increase in density.

In this study, we have developed QPID, a microfluidic platform for quantifying protein–DNA interactions in high throughput. The platform enables the measurement of thousands of experiments in parallel and up to four different proteins on a single device, in four parallel and independently activated sections. This is effectively 1–2 orders of magnitude more than EMSA based methods. QPID also has significant advantages over current methods in term of quantitative affinity measurements. QPID's sensitivity is in the low micromolar range. Its programmable nature allows performing large screens for de-novo characterization of DNA target sequences or alternatively many parallel small screens for quantitative measurement of protein–DNA interaction affinities. Last, microfluidic-based immune immobilization scheme provides flexibility. We can apply both *in vitro* expressed proteins and cellular extracts to QPID. Since we purify and concentrate proteins or complexes on-chip, we can work with difficult or low abundance proteins.

We plan to extend QPID in several ways. Currently, QPID measures affinity at equilibrium only. In the future, we will combine QPID with methodologies such as total internal reflection fluorescence microscopy. This will enable to directly measure the on/off rate kinetic parameters for the protein–DNA binding events. These kinetic parameters may provide new insights into protein–DNA binding. We can use this method to further probe complexes by adding protein competitors or, alternatively, analyze the combinatorial action of several transcription factors by introducing multiple binding sites on each oligonucleotide. QPID may also be applied to conservation and evolutionary studies, such as analyzing the importance of specific changes in DNA BS observed for the same TF in different organisms. In addition, QPID can be used to answer affinity questions raised by gcPBMs. Taking specificity models learned by gcPBMs, a library of representative oligos from the gcPBM experiment may be chosen and used by QPID in order to enhance the models with affinity measurements.

QPID can serve as an important research tool for protein DNA-binding. Many aspects of *in vivo* binding are still unknown, such as why some putative binding sites are unbound. By accurate measurement of *K*_d_ values of thousands of DNA oligonucleotides, the mechanism behind sequence-specific binding may be revealed. More importantly, the effect of local flanking sequences near the core binding element can be measured in high accuracy and enable the inference of new computational models for protein–DNA binding. This will improve our ability to predict protein–DNA binding at higher accuracy and distinguish between BSs of proteins from the same family. Given its advantages over extant techniques and its high throughput, we expect QPID to become a useful and valuable tool for studying protein–DNA interactions.

## Supplementary Material

SUPPLEMENTARY DATA
