# Supplementary material for: Integrated microfluidic approach for quantitative high-throughput measurements of transcription factor binding affinities
Source: Nucleic Acids Res. 2015 Dec 3;44(6):e51. doi: 10.1093/nar/gkv1327 (PMC4824076; doi:10.1093/nar/gkv1327)
Supplement: SUPPLEMENTARY DATA [file supp_44_6_e51__index.html]

Integrated microfluidic approach for quantitative high-throughput measurements of transcription factor binding affinities — Integrated microfluidic approach for quantitative high-throughput measurements of transcription factor binding affinities — SUPPLEMENTARY DATA 

# Integrated microfluidic approach for quantitative high-throughput measurements of transcription factor binding affinities

## SUPPLEMENTARY DATA

- SUPPLEMENTARY DATA
- SUPPLEMENTARY DATA
- SUPPLEMENTARY DATA
- SUPPLEMENTARY DATA
- SUPPLEMENTARY DATA
