# Supplementary material for: Integrated microfluidic approach for quantitative high-throughput measurements of transcription factor binding affinities
Source: Nucleic Acids Res. 2015 Dec 3;44(6):e51. doi: 10.1093/nar/gkv1327 (PMC4824076; doi:10.1093/nar/gkv1327)
Supplement: SUPPLEMENTARY DATA [file supp_gkv1327_nar-00752-met-k-2015-File003.pdf]

Supplementary Figures

# Integrated Microfluidic Approach For Quantitative High-Throughput Measurements Of Transcription Factor Binding Affinities

Yair Glick[1,4], Yaron Orenstein[2,4], Dana Chen[1], Dorit Avrahami[1], Tsaffrir Zor[3], Ron Shamir[2], Doron Gerber[1*]

Mina and Evrard Goodman life science faculty, Bar Ilan University, Ramat-Gan, 5290002, Israel

Blavatnik School of Computer Science, Tel-Aviv University, Tel-Aviv, 69978, Israel

Department of Biochemistry & Molecular Biology, Life Sciences Institute, Tel-Aviv University, Tel-Aviv, 69978, Israel

The authors contributed equally to this work.

* To whom correspondence should be addressed.

Tel: +972 3 7384508; Fax: +942 3 7384196; Email: Doron.Gerber@biu.ac.il

Fig. S1

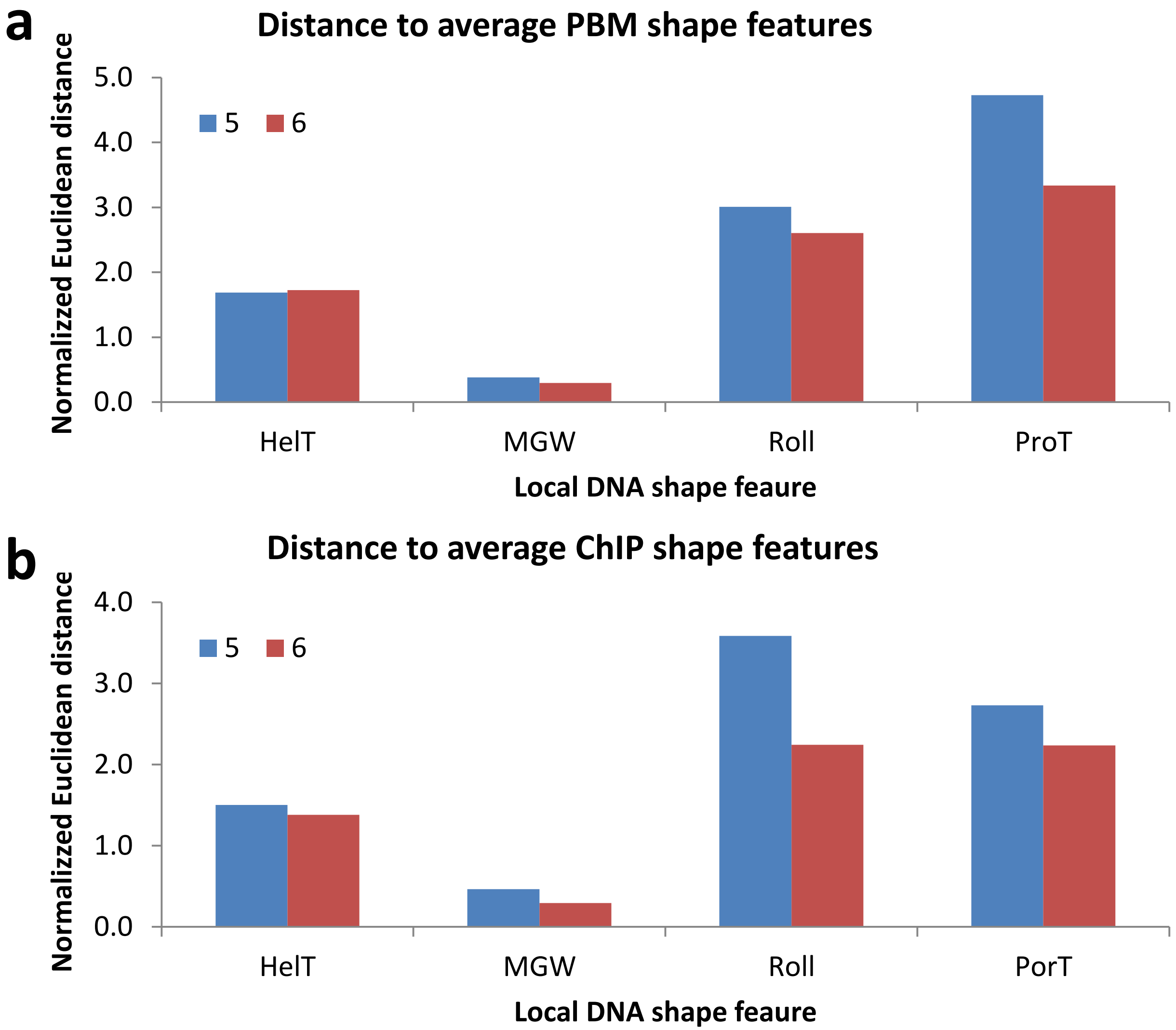


**Figure S1 - Similarity of QPID ATF1 binding preferences to PBM and ChIP-seq.** Normalized Euclidean distances were calculated between DNA local shape feature predictions of QPID oligonucleotides 5 and 6 and average shape feature predictions of CRE half-sites binding sites (TGACG) (see Methods). **(a)** Distance to an average of 1619 BSs measured by PBM. **(b)** Distance to an average of 7545 BSs measured by ChIP-seq. HelT: helix twist, MGW: minor groove width, ProT: propeller twist.

Fig. S2

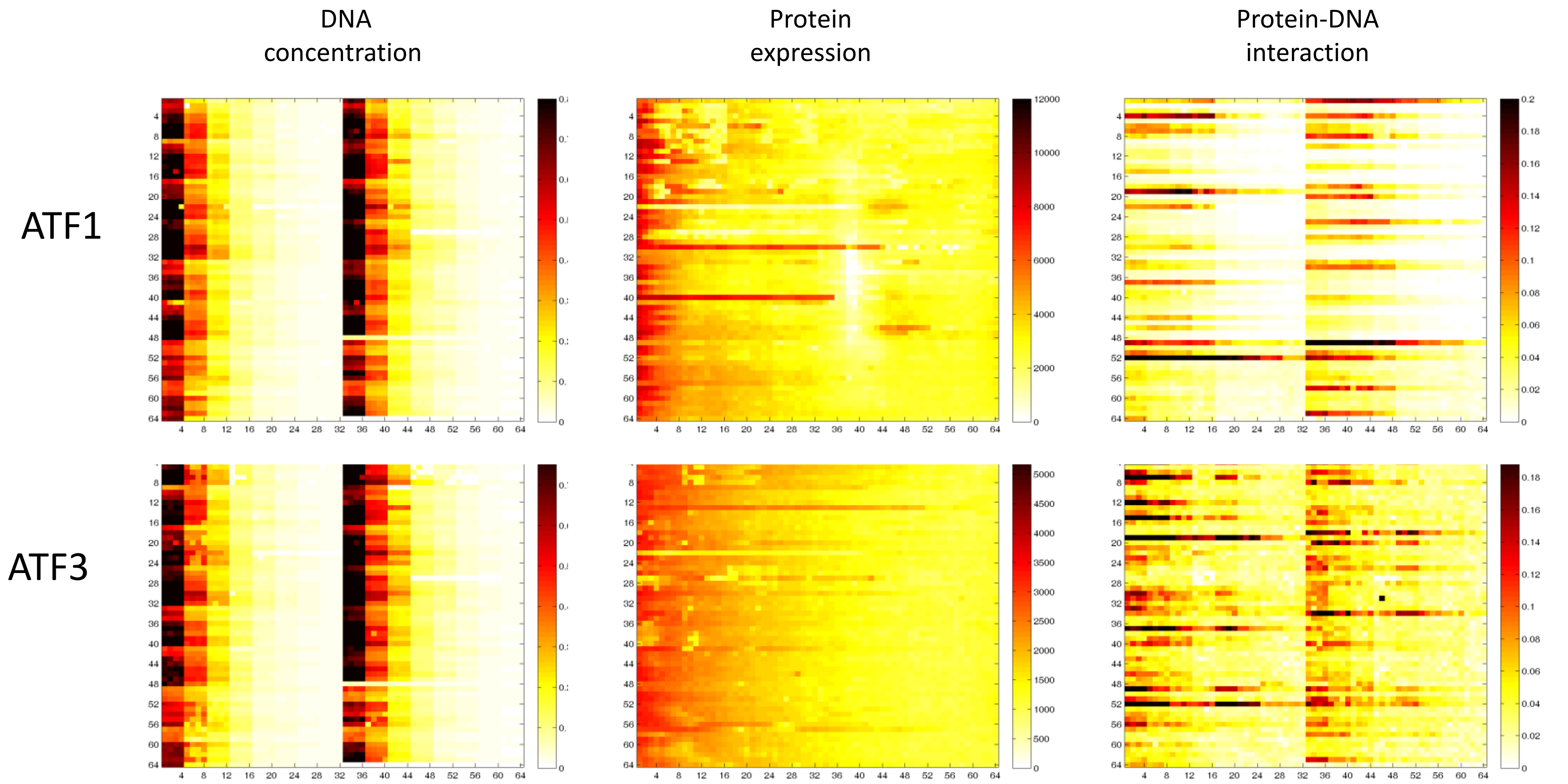


**Figure S2 - Heat maps of ATF1 and ATF3 QPID analysis**. DNA concentration (µM) in solution for the various oligonucleotides is shown on the left panel. Protein signals are shown in the middle panel (fluorescent intensity, arbitrary units). DNA binding to the protein complexes is shown in the right panel.

Supplementary Figure S3

**Figure S3 - ATF1 Quantitative analysis Kd's.** Affinity measurements between ATF1 and CRE sequences. We programmed the QPID device with different genomic CRE sequences. ATF1 was immobilized to the device surface, flooding DNA chambers solubilized spotted DNA, allowing ATF1 and DNA to interact. Protein expression levels (Cy3) and interacting DNA signals (Cy5) were measured. Interaction ratio – Cy5/Cy3

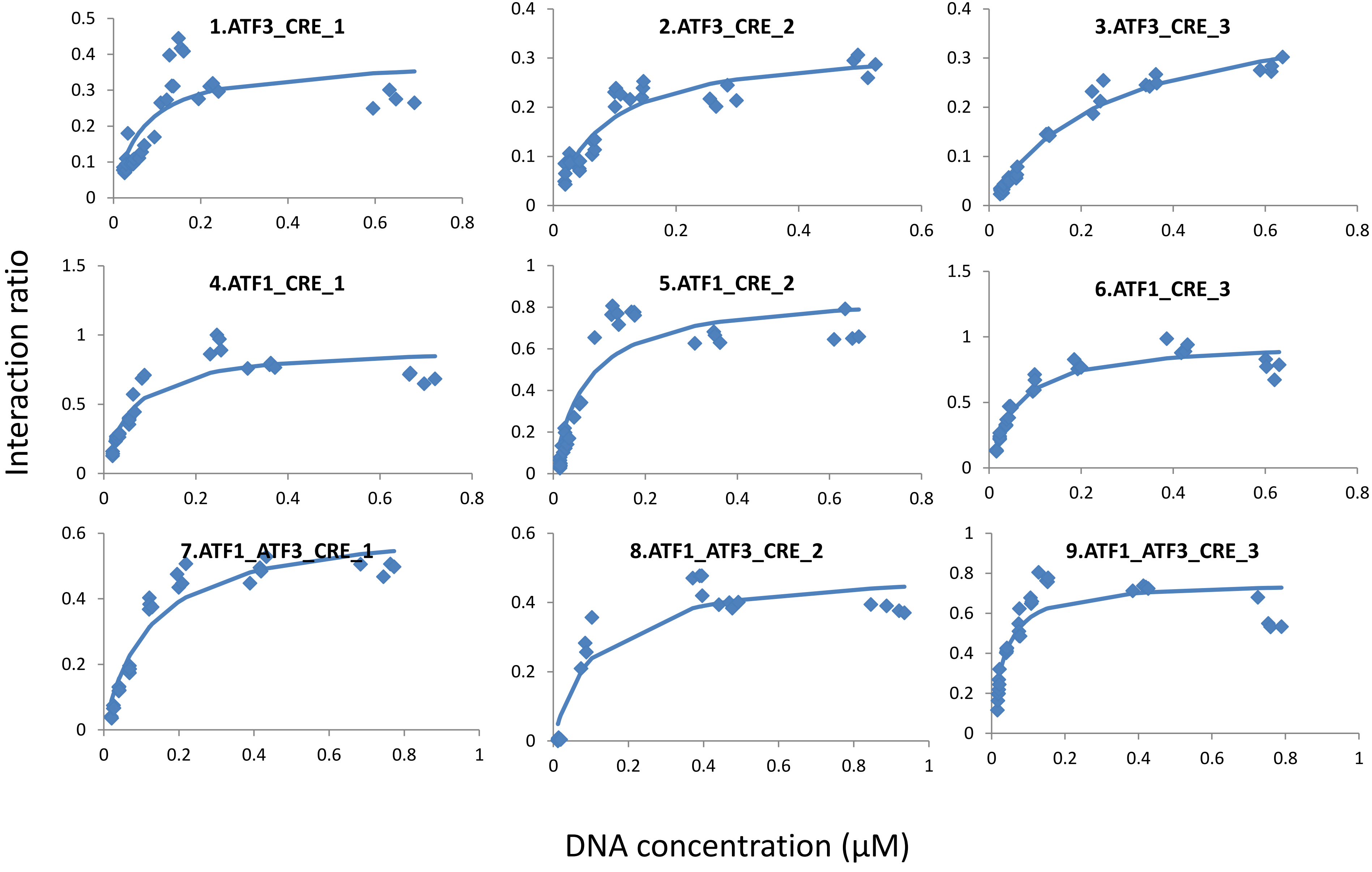

Interaction ratio
1.ATF3_CRE_1
2.ATF3_CRE_2
3.ATF3_CRE_3
4.ATF1_CRE_1
5.ATF1_CRE_2
6.ATF1_CRE_3
7.ATF1_ATF3_CRE_1
8.ATF1_ATF3_CRE_2
9.ATF1_ATF3_CRE_3
DNA concentration (μM)

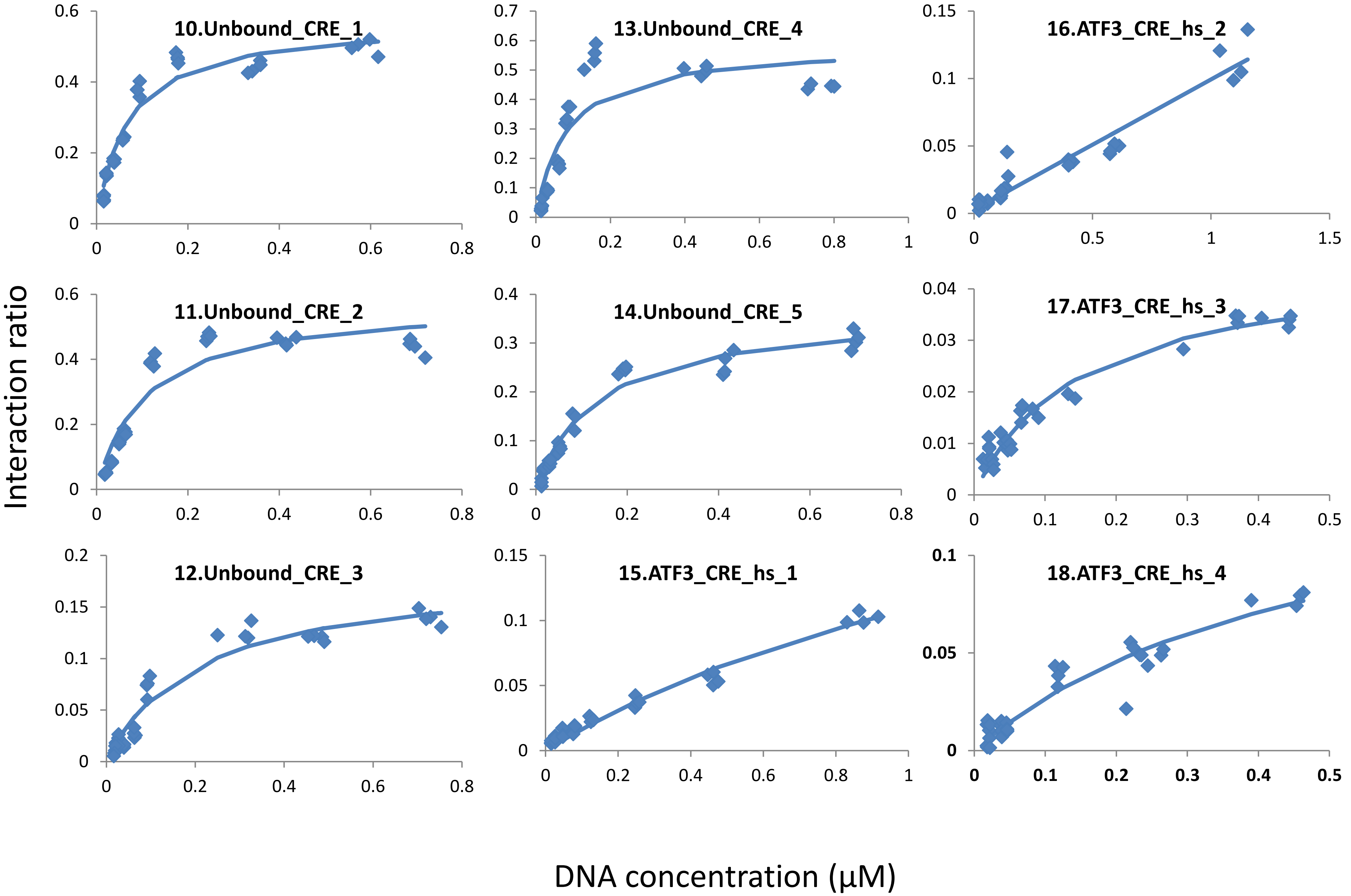

10.Unbound_CRE_1
13.Unbound_CRE_4
16.ATF3_CRE_hs_2
11.Unbound_CRE_2
14.Unbound_CRE_5
17.ATF3_CRE_hs_3
12.Unbound_CRE_3
15.ATF3_CRE_hs_1
18.ATF3_CRE_hs_4
Interaction ratio
DNA concentration (μM)

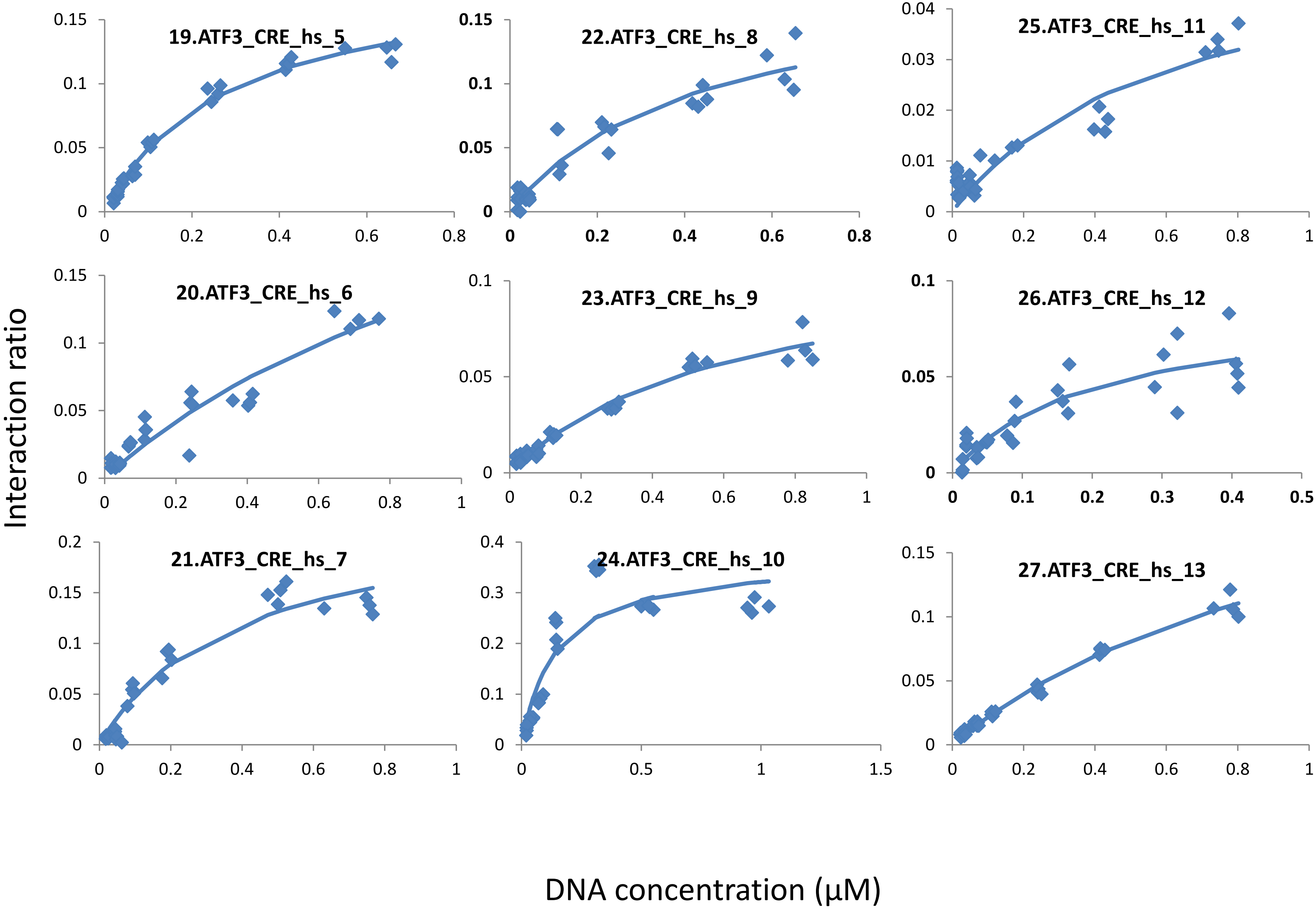

19.ATF3_CRE_hs_5
22.ATF3_CRE_hs_8
25.ATF3_CRE_hs_11
20.ATF3_CRE_hs_6
23.ATF3_CRE_hs_9
26.ATF3_CRE_hs_12
21.ATF3_CRE_hs_7
24.ATF3_CRE_hs_10
27.ATF3_CRE_hs_13
Interaction ratio
DNA concentration (μM)

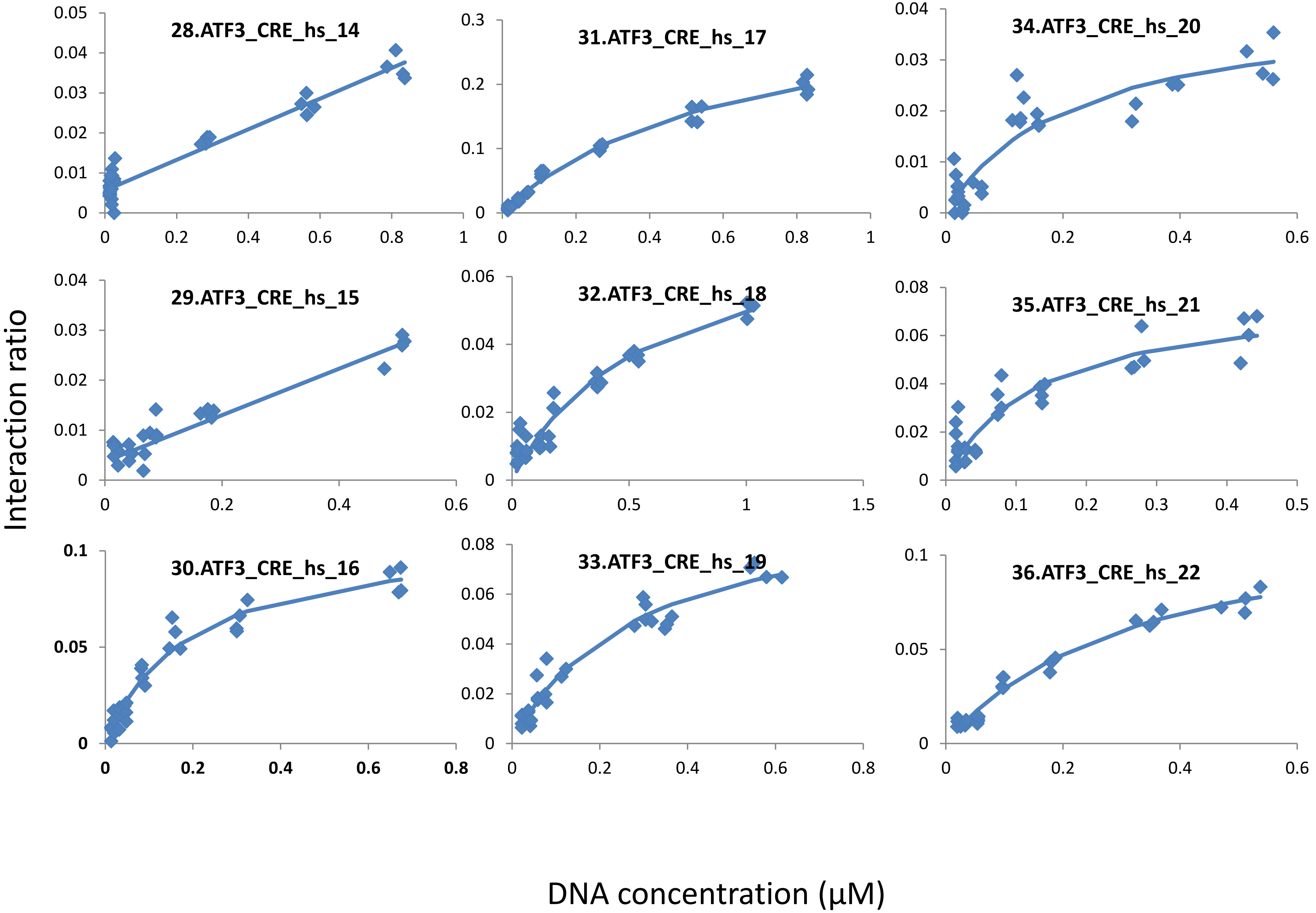
28.ATF3_CRE_hs_14
31.ATF3_CRE_hs_17
34.ATF3_CRE_hs_20
29.ATF3_CRE_hs_15
32.ATF3_CRE_hs_18
35.ATF3_CRE_hs_21
30.ATF3_CRE_hs_16
33.ATF3_CRE_hs_19
36.ATF3_CRE_hs_22
Interaction ratio
DNA concentration (μM)

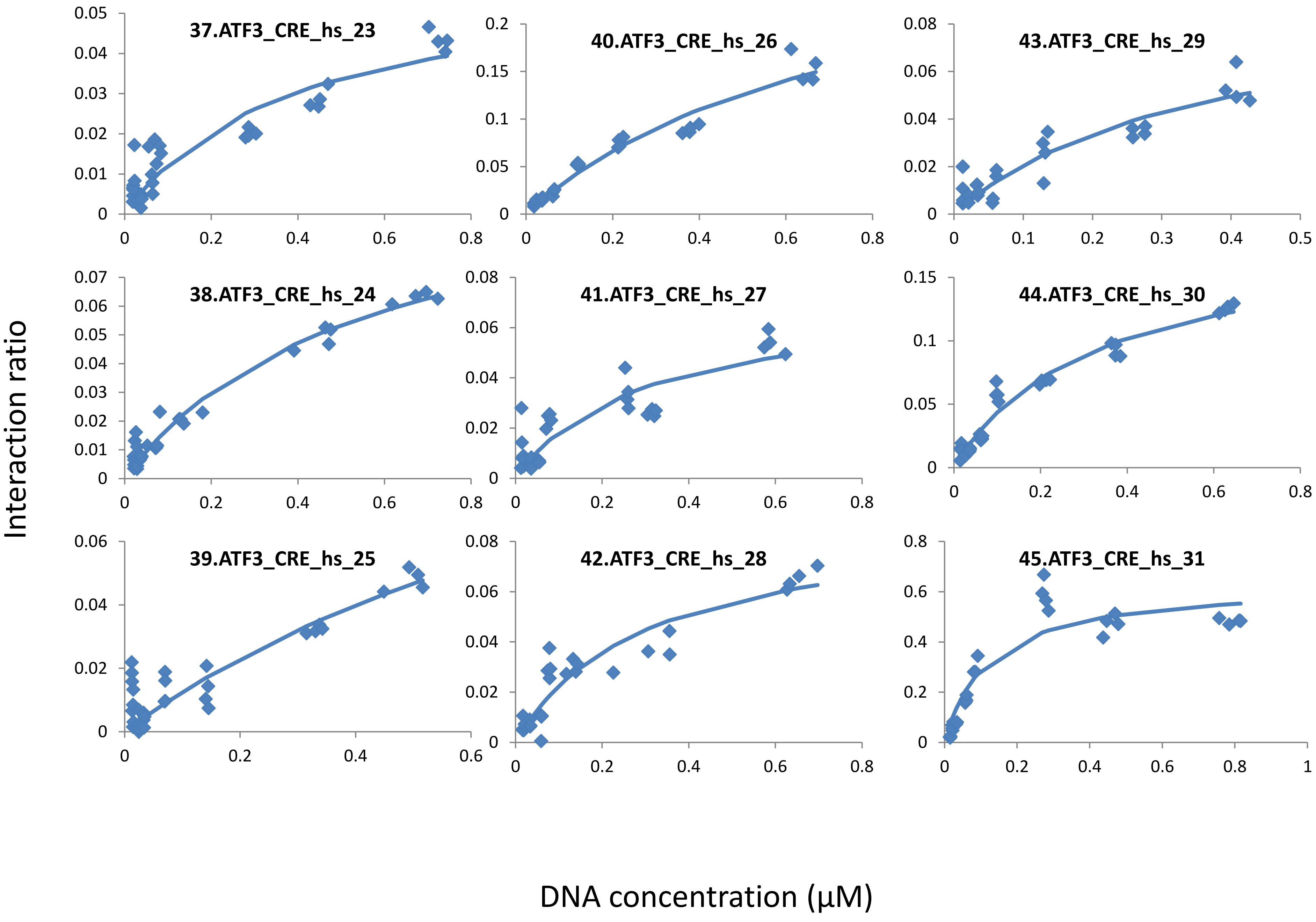

37.ATF3_CRE_hs_23
40.ATF3_CRE_hs_26
43.ATF3_CRE_hs_29
38.ATF3_CRE_hs_24
41.ATF3_CRE_hs_27
44.ATF3_CRE_hs_30
39.ATF3_CRE_hs_25
42.ATF3_CRE_hs_28
45.ATF3_CRE_hs_31
Interaction ratio
DNA concentration (μM)

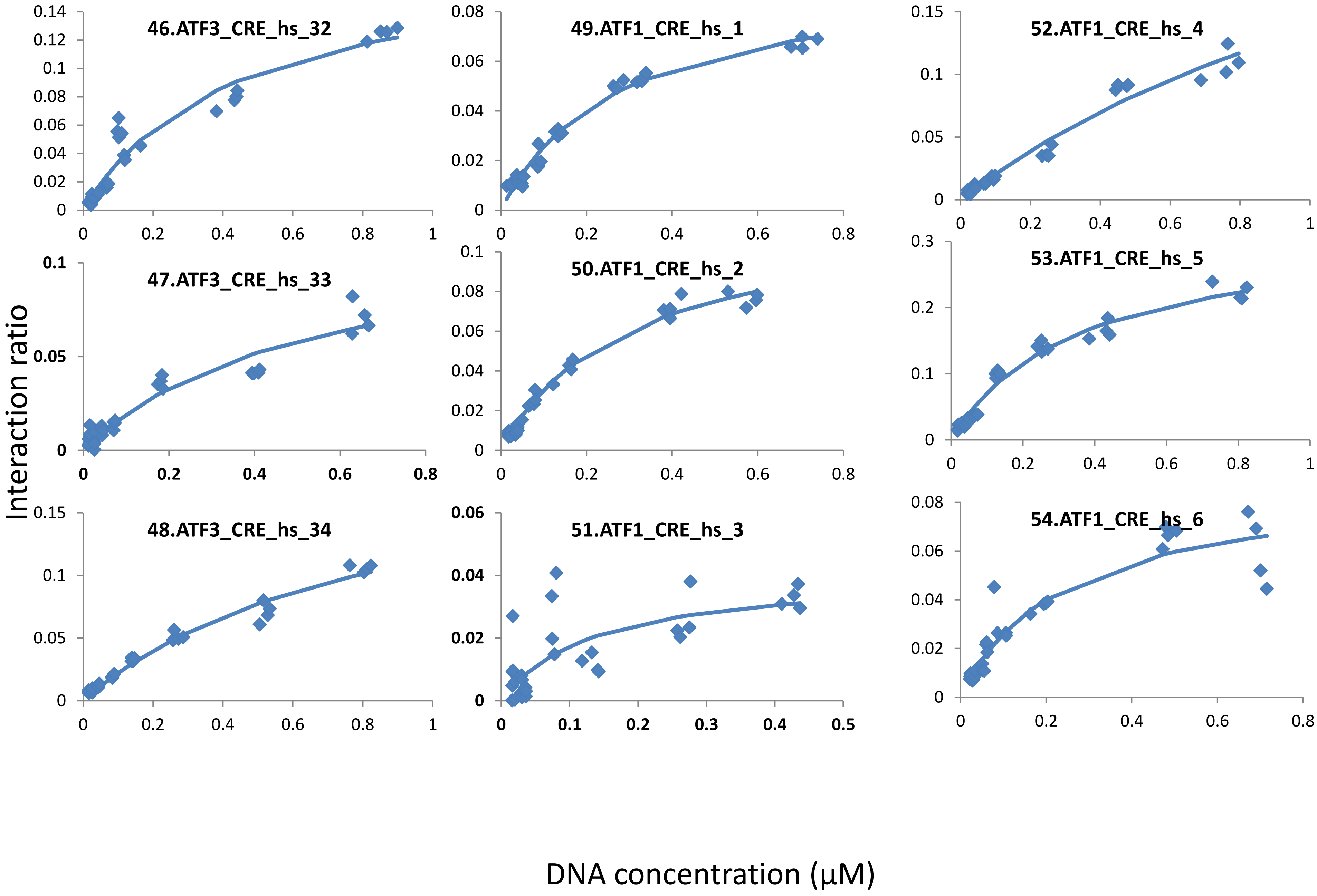
46.ATF3_CRE_hs_32
49.ATF1_CRE_hs_1
52.ATF1_CRE_hs_4
47.ATF3_CRE_hs_33
50.ATF1_CRE_hs_2
53.ATF1_CRE_hs_5
48.ATF3_CRE_hs_34
51.ATF1_CRE_hs_3
54.ATF1_CRE_hs_6
Interaction ratio
DNA concentration (μM)

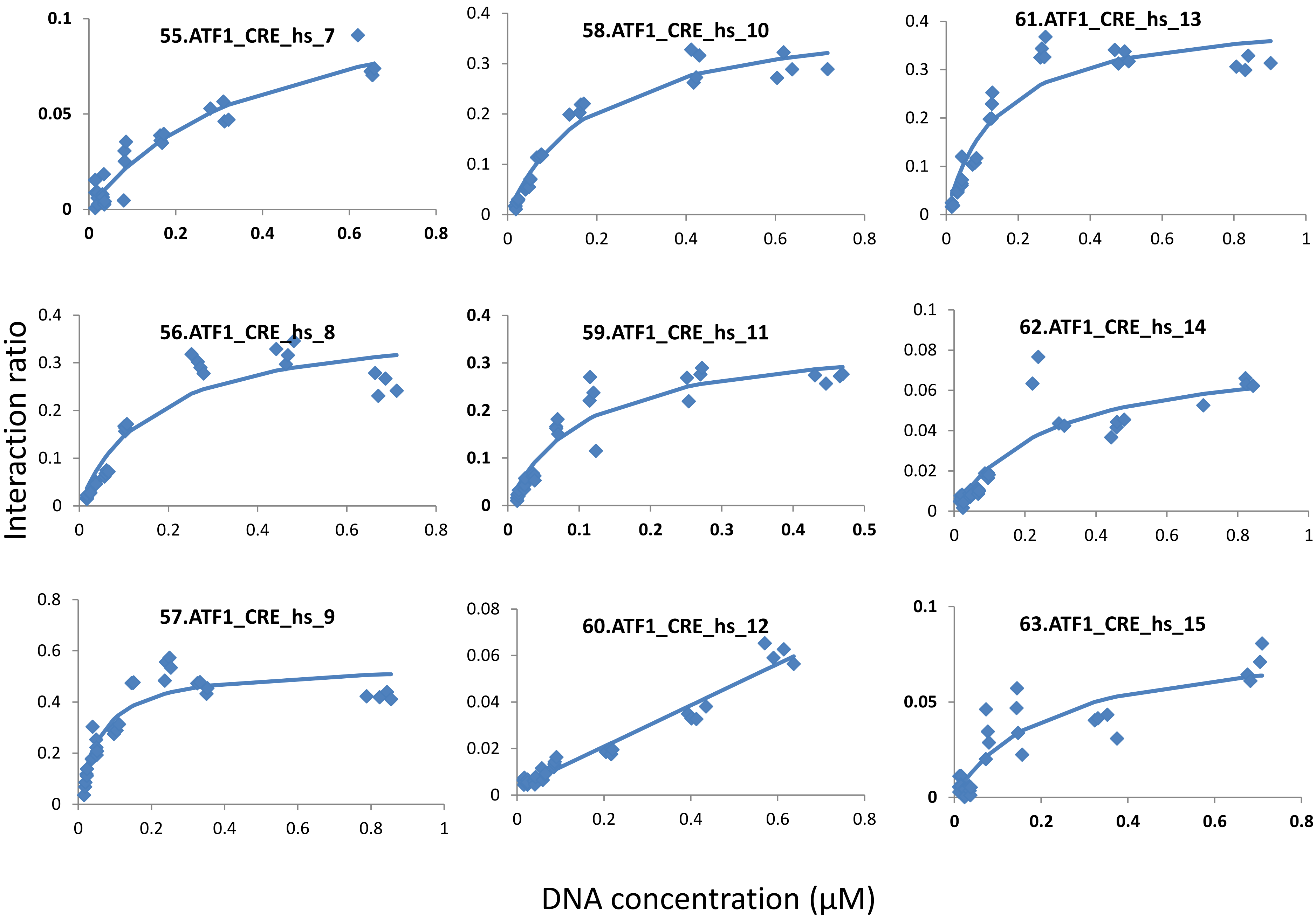
55.ATF1_CRE_hs_7
58.ATF1_CRE_hs_10
61.ATF1_CRE_hs_13
56.ATF1_CRE_hs_8
59.ATF1_CRE_hs_11
62.ATF1_CRE_hs_14
57.ATF1_CRE_hs_9
60.ATF1_CRE_hs_12
63.ATF1_CRE_hs_15
Interaction ratio
DNA concentration (μM)

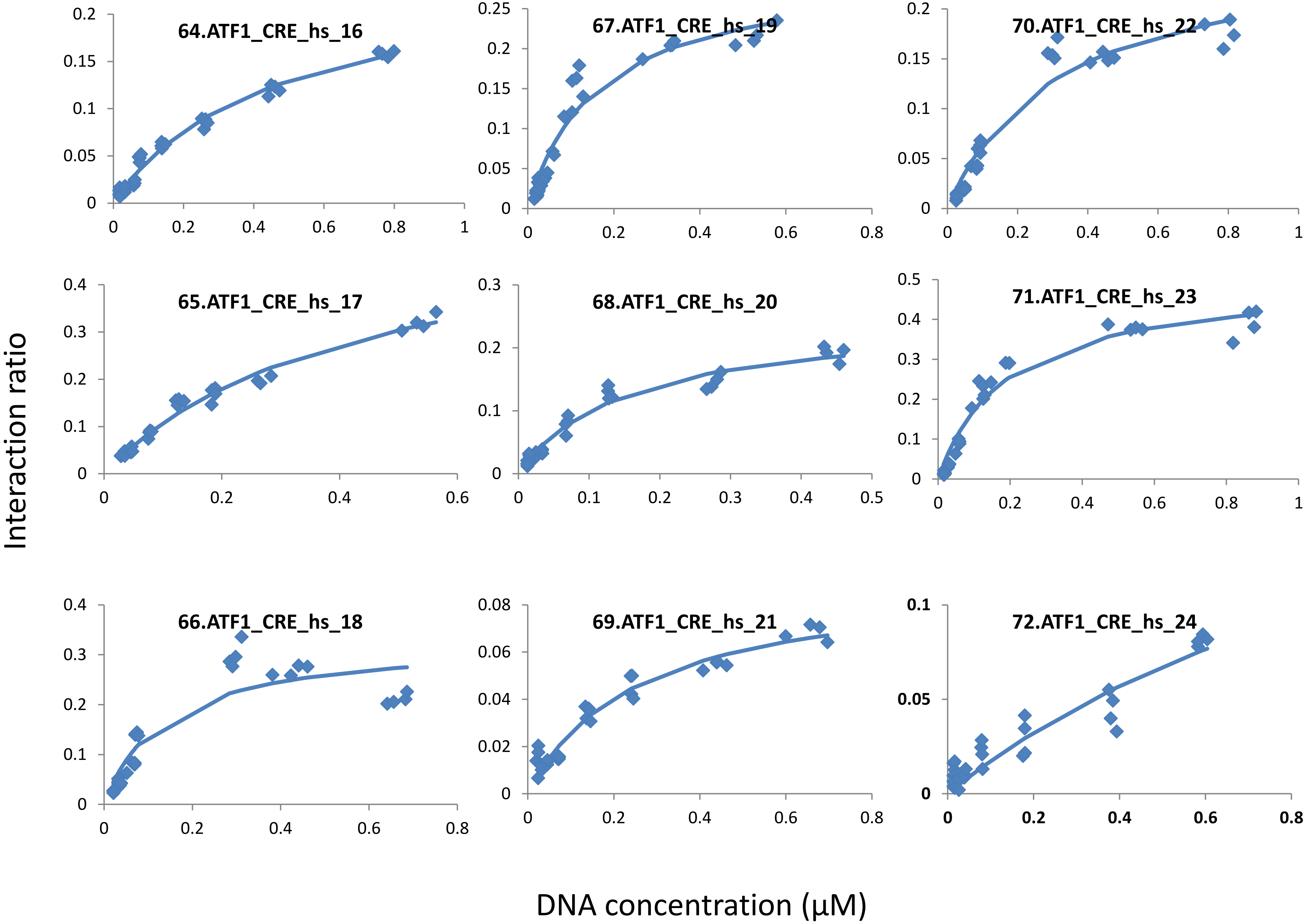
64.ATF1_CRE_hs_16
67.ATF1_CRE_hs_19
70.ATF1_CRE_hs_22
65.ATF1_CRE_hs_17
68.ATF1_CRE_hs_20
71.ATF1_CRE_hs_23
66.ATF1_CRE_hs_18
69.ATF1_CRE_hs_21
72.ATF1_CRE_hs_24
Interaction ratio
DNA concentration (μM)

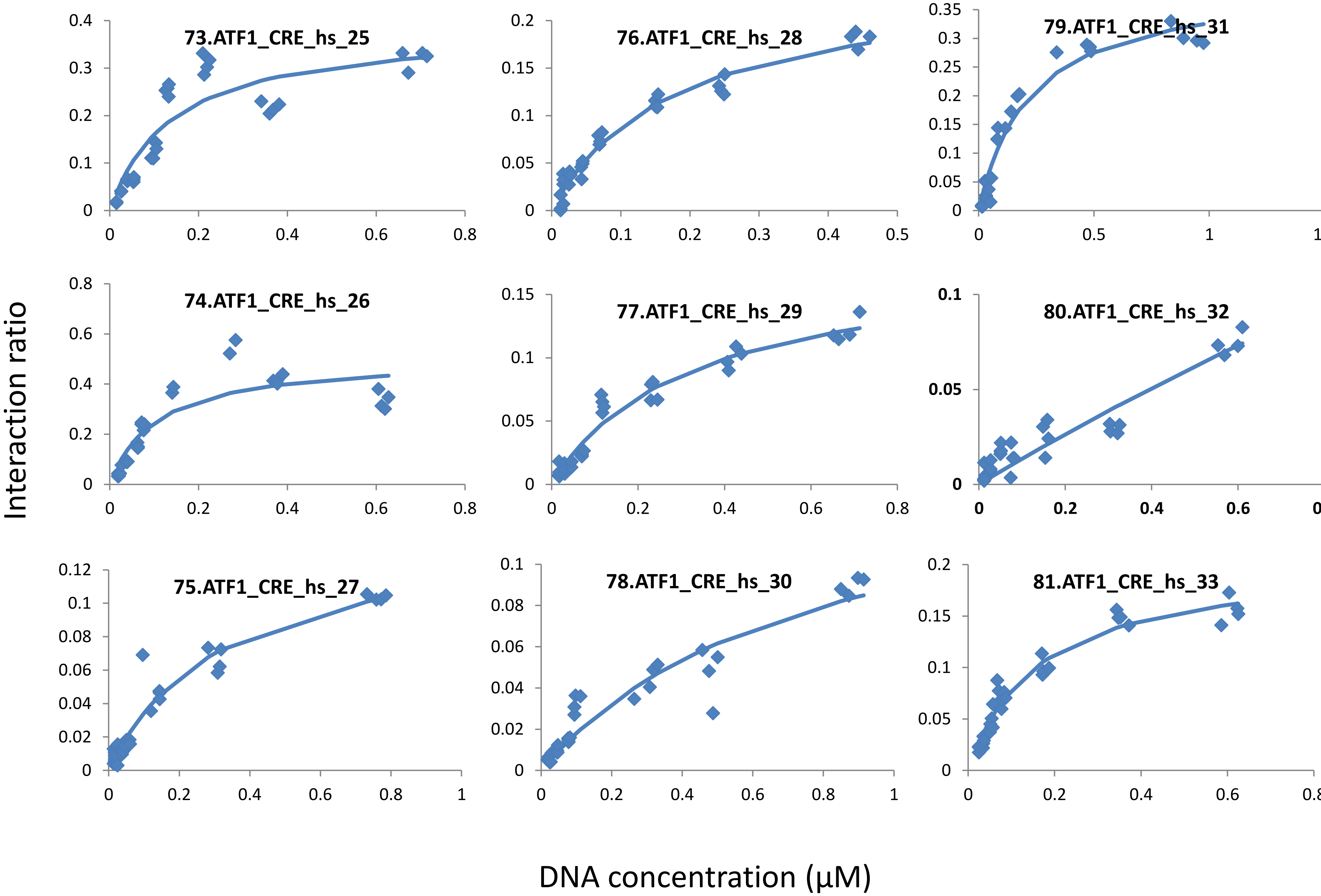

73.ATF1_CRE_hs_25
76.ATF1_CRE_hs_28
79.ATF1_CRE_hs_31
74.ATF1_CRE_hs_26
77.ATF1_CRE_hs_29
80.ATF1_CRE_hs_32
75.ATF1_CRE_hs_27
78.ATF1_CRE_hs_30
81.ATF1_CRE_hs_33
Interaction ratio
DNA concentration (μM)

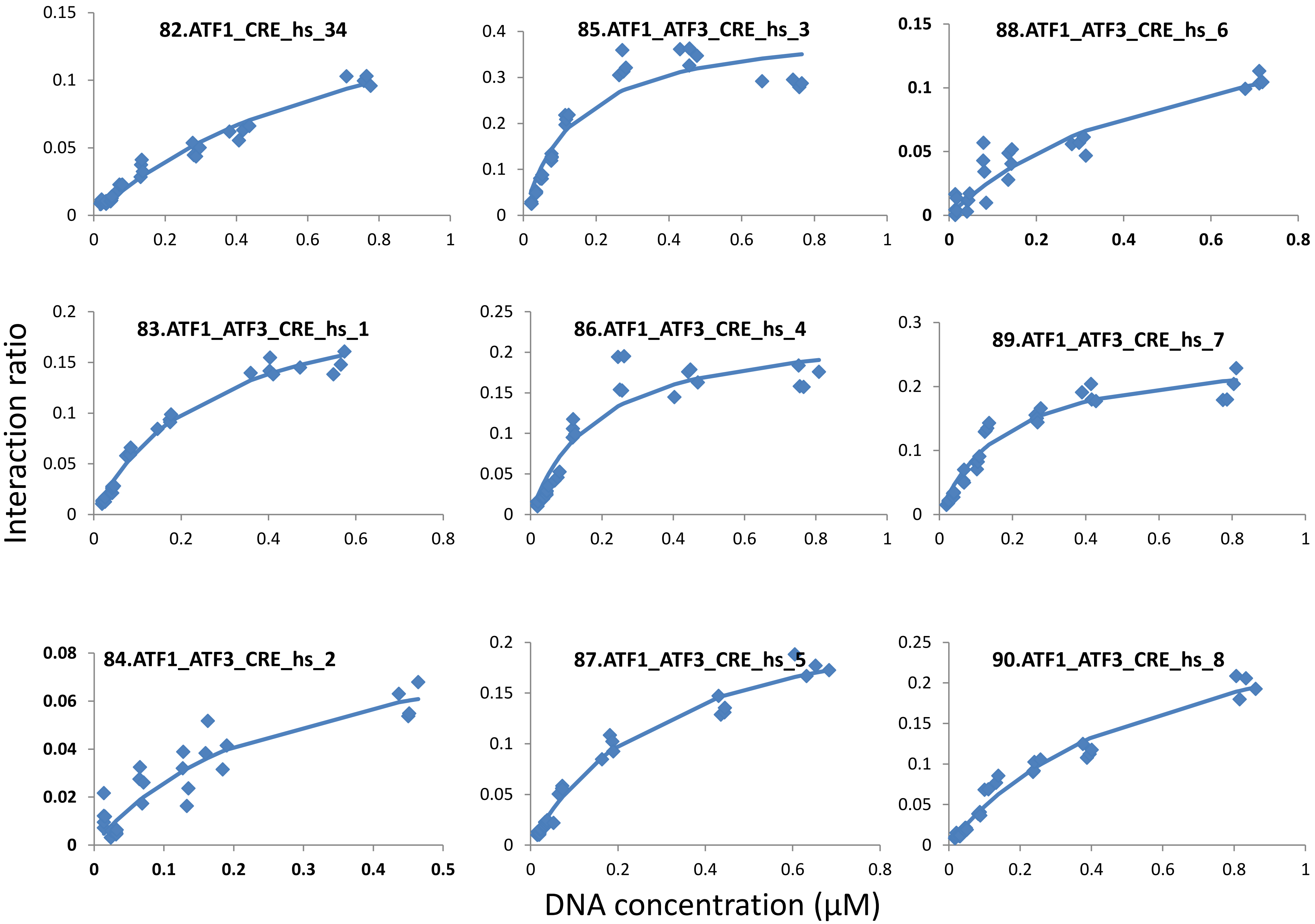
82.ATF1_CRE_hs_34
85.ATF1_ATF3_CRE_hs_3
88.ATF1_ATF3_CRE_hs_6
83.ATF1_ATF3_CRE_hs_1
86.ATF1_ATF3_CRE_hs_4
89.ATF1_ATF3_CRE_hs_7
84.ATF1_ATF3_CRE_hs_2
87.ATF1_ATF3_CRE_hs_5
90.ATF1_ATF3_CRE_hs_8
Interaction ratio
DNA concentration (μM)

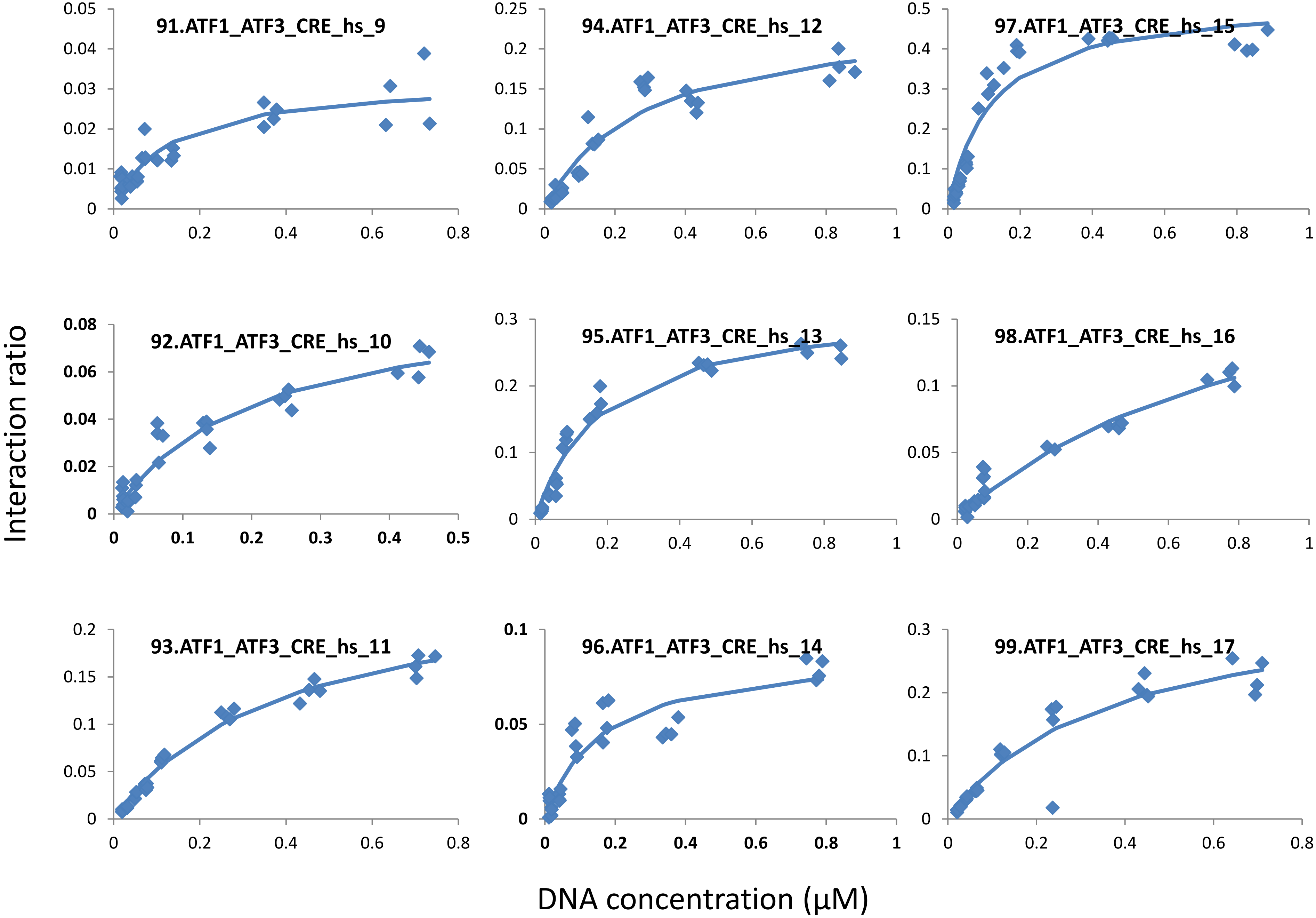
91.ATF1_ATF3_CRE_hs_9
94.ATF1_ATF3_CRE_hs_12
97.ATF1_ATF3_CRE_hs_15
92.ATF1_ATF3_CRE_hs_10
95.ATF1_ATF3_CRE_hs_13
98.ATF1_ATF3_CRE_hs_16
93.ATF1_ATF3_CRE_hs_11
96.ATF1_ATF3_CRE_hs_14
99.ATF1_ATF3_CRE_hs_17
Interaction ratio
DNA concentration (μM)

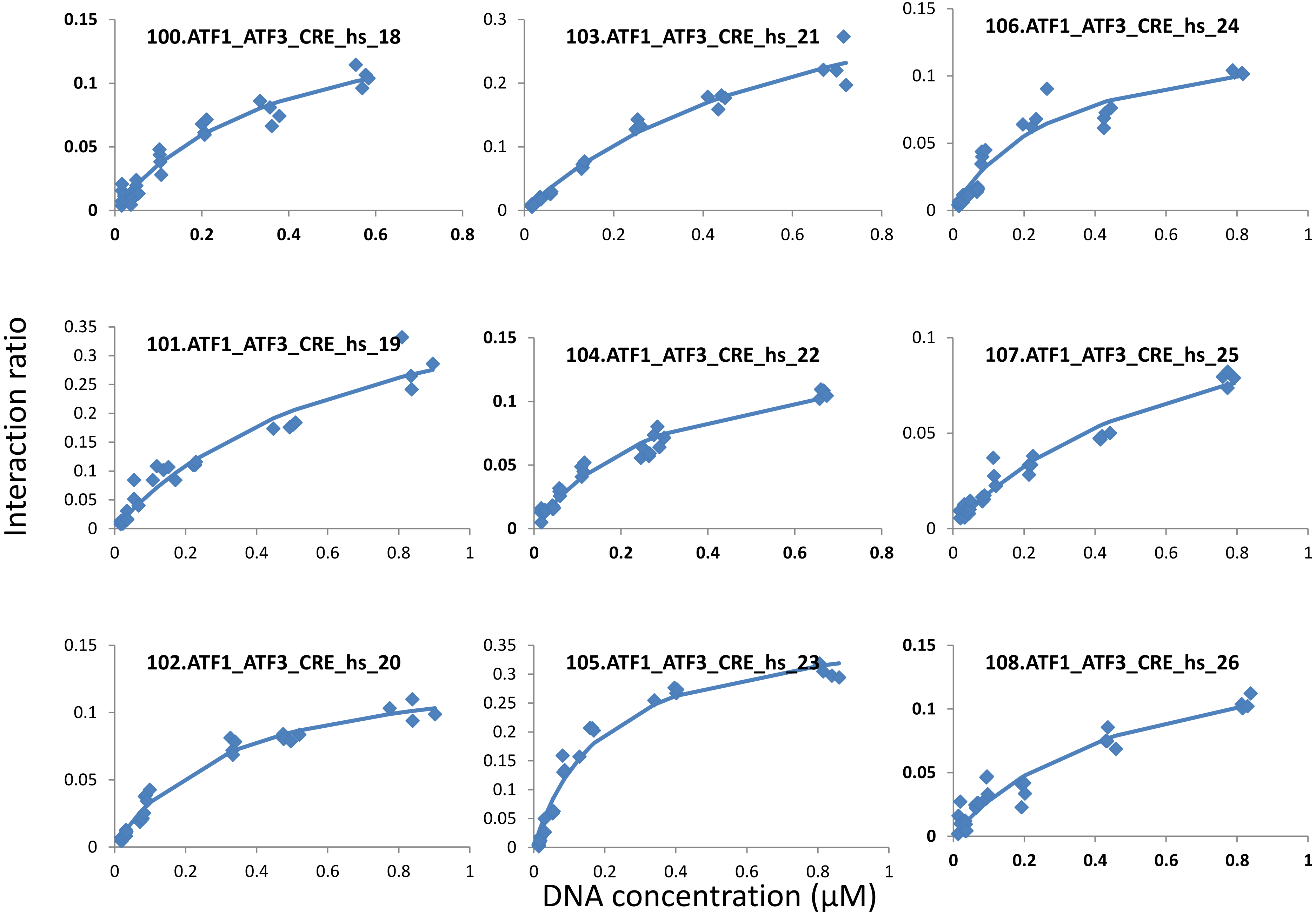
100.ATF1_ATF3_CRE_hs_18
103.ATF1_ATF3_CRE_hs_21
106.ATF1_ATF3_CRE_hs_24
101.ATF1_ATF3_CRE_hs_19
104.ATF1_ATF3_CRE_hs_22
107.ATF1_ATF3_CRE_hs_25
102.ATF1_ATF3_CRE_hs_20
105.ATF1_ATF3_CRE_hs_23
108.ATF1_ATF3_CRE_hs_26
Interaction ratio
DNA concentration (μM)

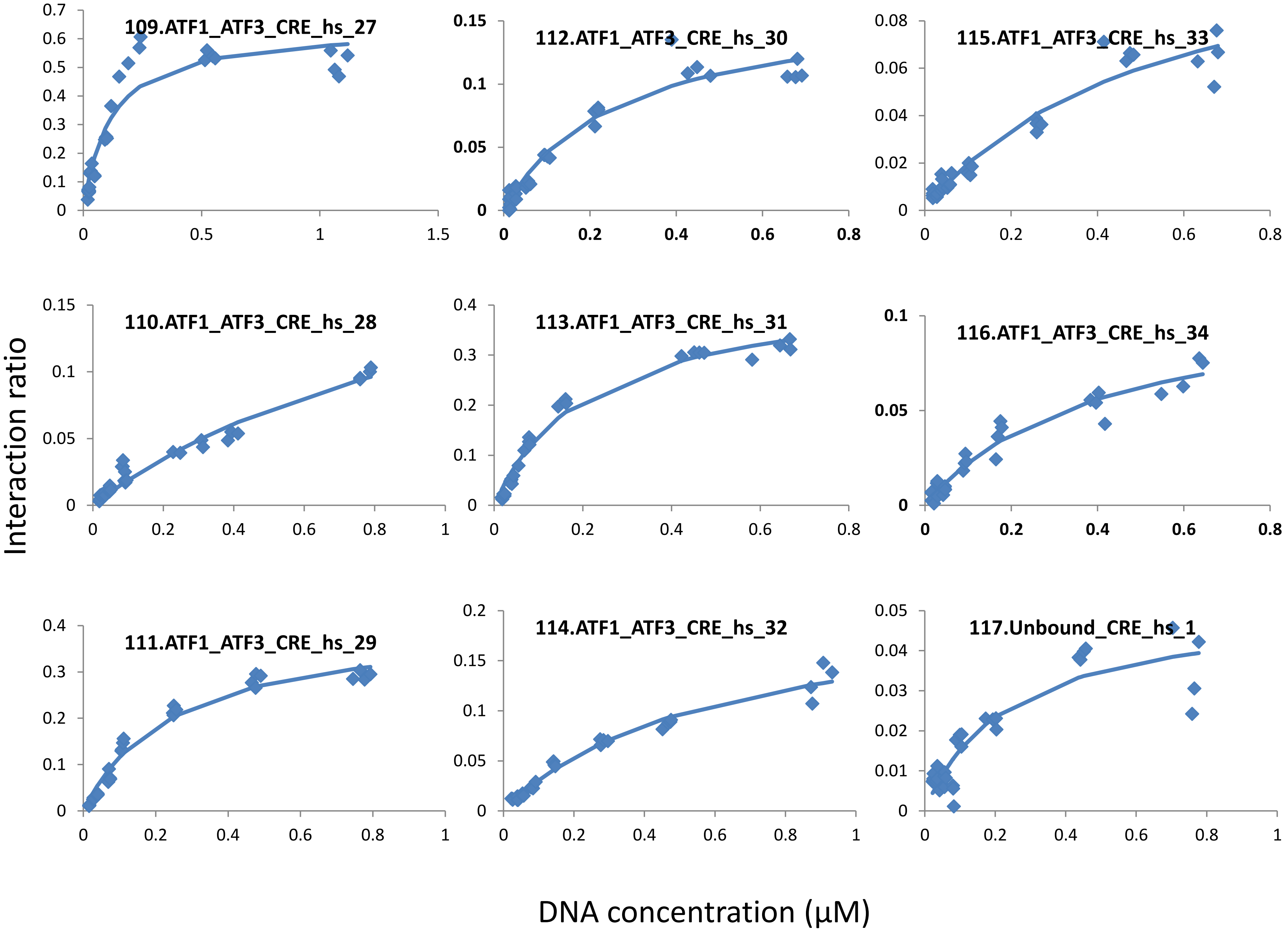
109.ATF1_ATF3_CRE_hs_27
112.ATF1_ATF3_CRE_hs_30
115.ATF1_ATF3_CRE_hs_33
110.ATF1_ATF3_CRE_hs_28
113.ATF1_ATF3_CRE_hs_31
116.ATF1_ATF3_CRE_hs_34
111.ATF1_ATF3_CRE_hs_29
114.ATF1_ATF3_CRE_hs_32
117.Unbound_CRE_hs_1
Interaction ratio
DNA concentration (μM)

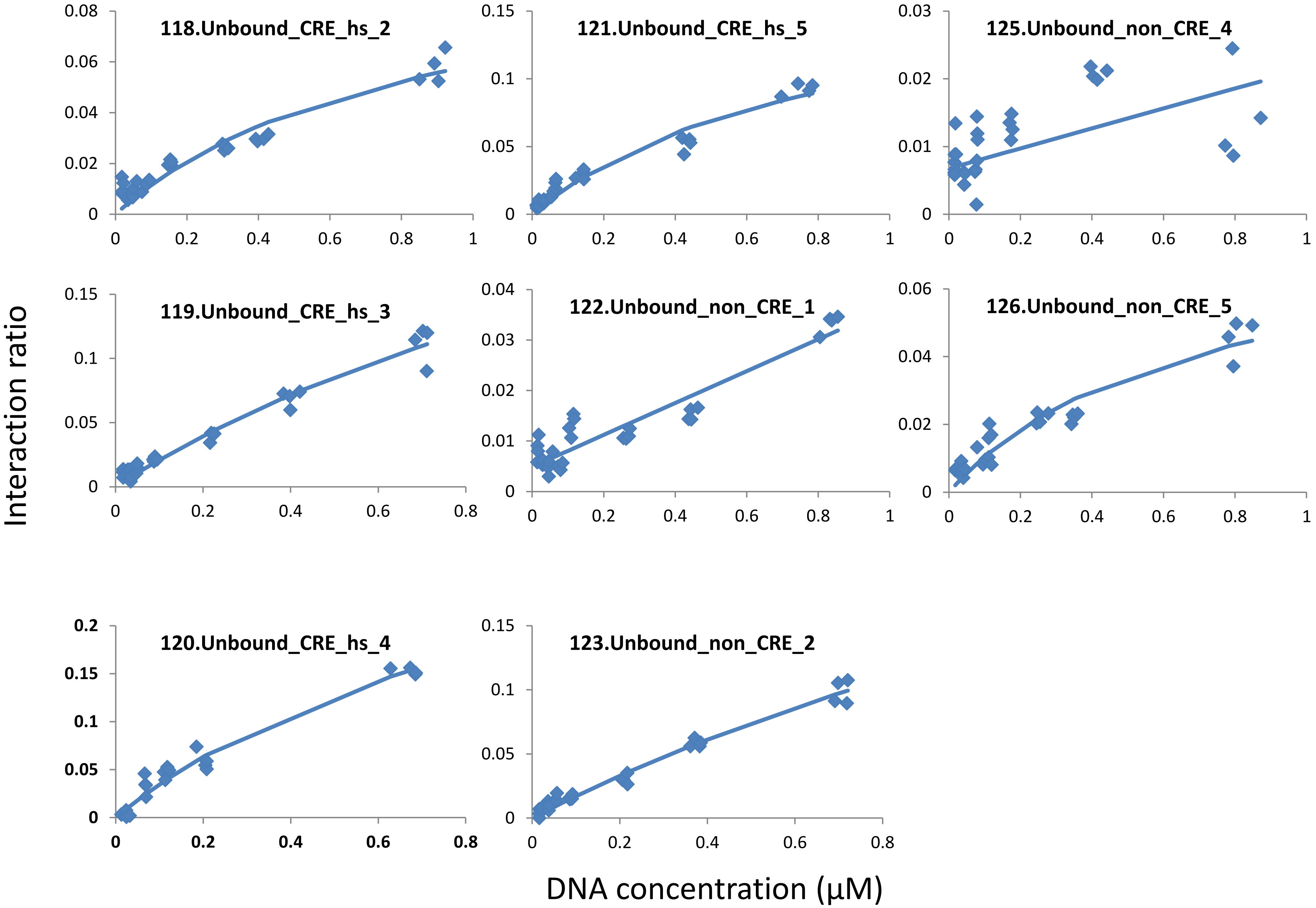
118.Unbound_CRE_hs_2
121.Unbound_CRE_hs_5
125.Unbound_non_CRE_4
119.Unbound_CRE_hs_3
122.Unbound_non_CRE_1
126.Unbound_non_CRE_5
120.Unbound_CRE_hs_4
123.Unbound_non_CRE_2
Interaction ratio
DNA concentration (μM)

Supplementary Figure S4

**Figure S4 – ATF3 Quantitative analysis Kd's.** Affinity measurements between ATF3 and CRE sequences. We programmed the QPID device with different genomic CRE sequences. ATF3 was immobilized to the device surface, flooding DNA chambers solubilized spotted DNA, allowing ATF3 and DNA to interact. Protein expression levels (Cy3) and interacting DNA signals (Cy5) were measured. Interaction ratio – Cy5/Cy3

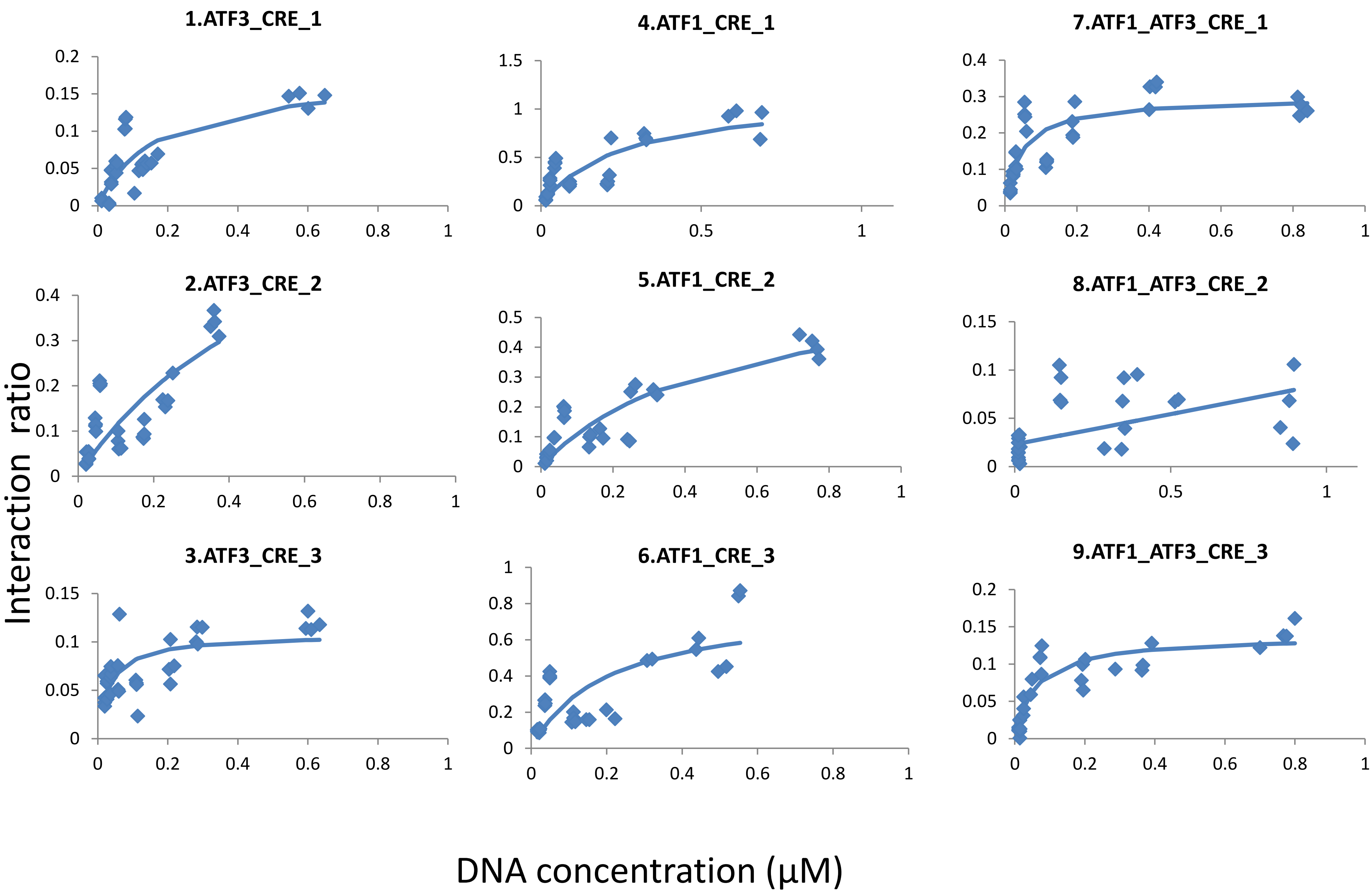
1.ATF3_CRE_1
2.ATF3_CRE_2
3.ATF3_CRE_3
4.ATF1_CRE_1
5.ATF1_CRE_2
6.ATF1_CRE_3
7.ATF1_ATF3_CRE_1
8.ATF1_ATF3_CRE_2
9.ATF1_ATF3_CRE_3
Interaction ratio
DNA concentration (µM)

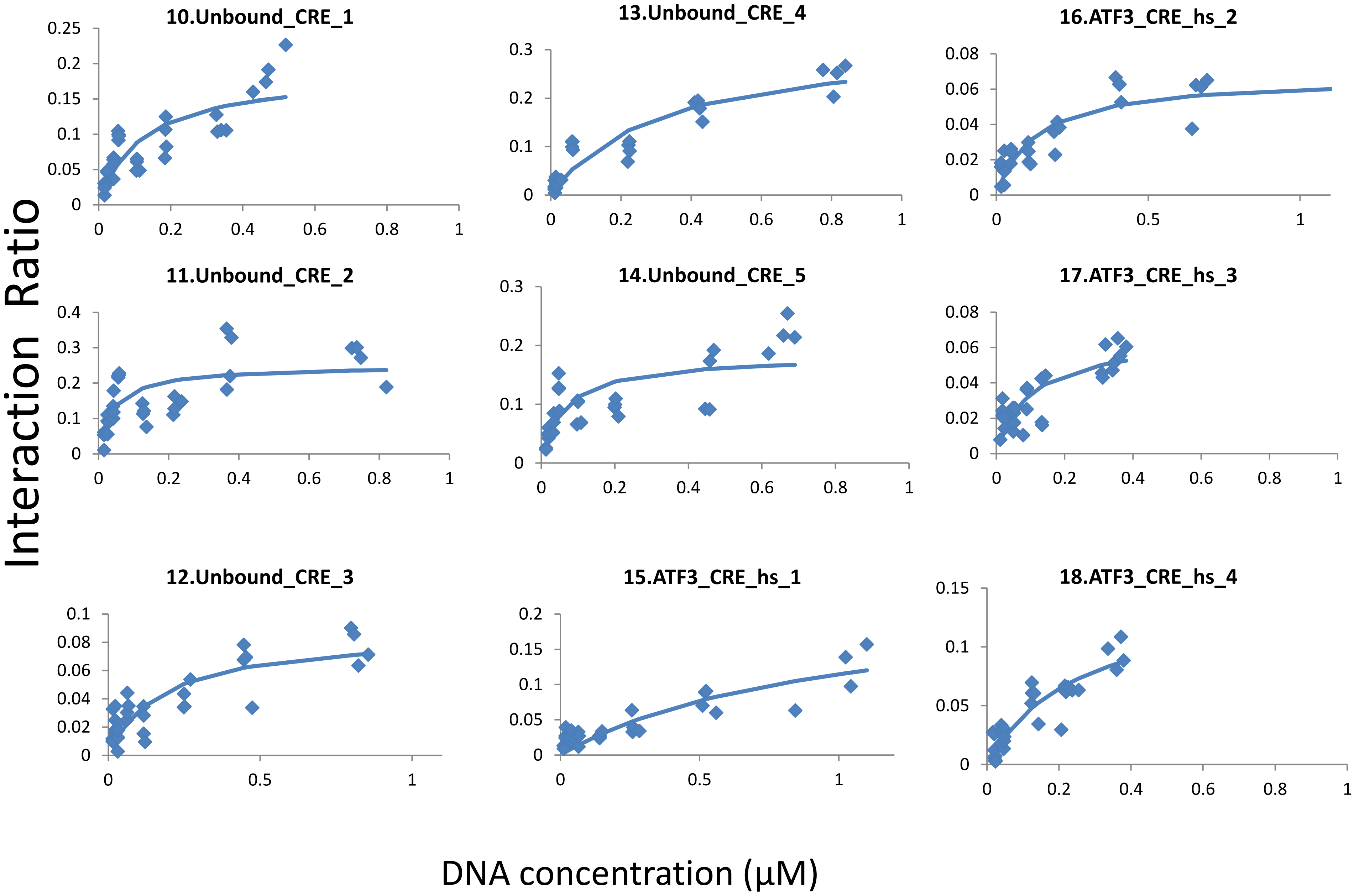

Interaction Ratio
10.Unbound_CRE_1
11.Unbound_CRE_2
12.Unbound_CRE_3
13.Unbound_CRE_4
14.Unbound_CRE_5
15.ATF3_CRE_hs_1
16.ATF3_CRE_hs_2
17.ATF3_CRE_hs_3
18.ATF3_CRE_hs_4
DNA concentration (µM)

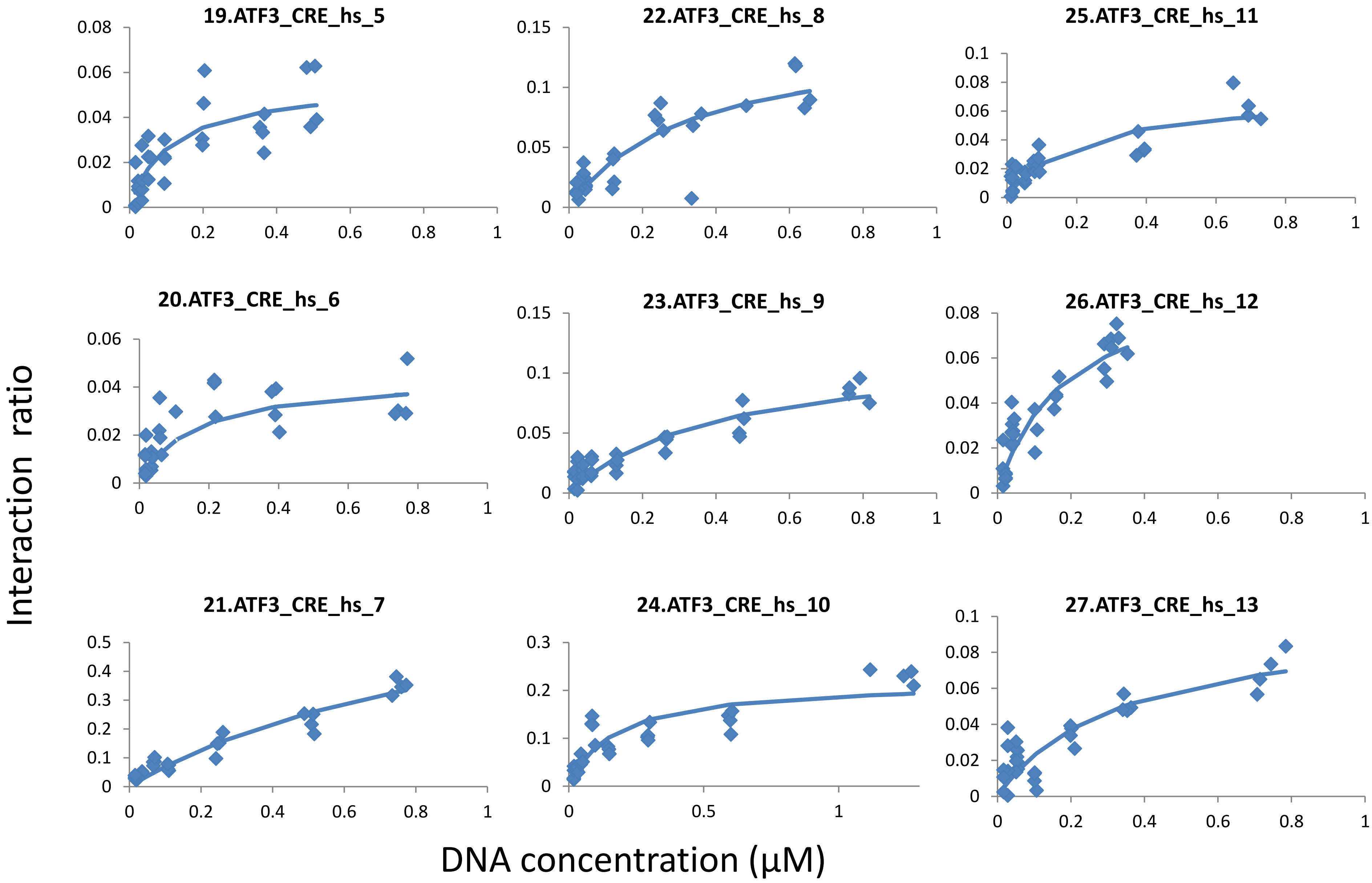

19.ATF3_CRE_hs_5
22.ATF3_CRE_hs_8
25.ATF3_CRE_hs_11
20.ATF3_CRE_hs_6
23.ATF3_CRE_hs_9
26.ATF3_CRE_hs_12
21.ATF3_CRE_hs_7
24.ATF3_CRE_hs_10
27.ATF3_CRE_hs_13
Interaction ratio
DNA concentration (μM)

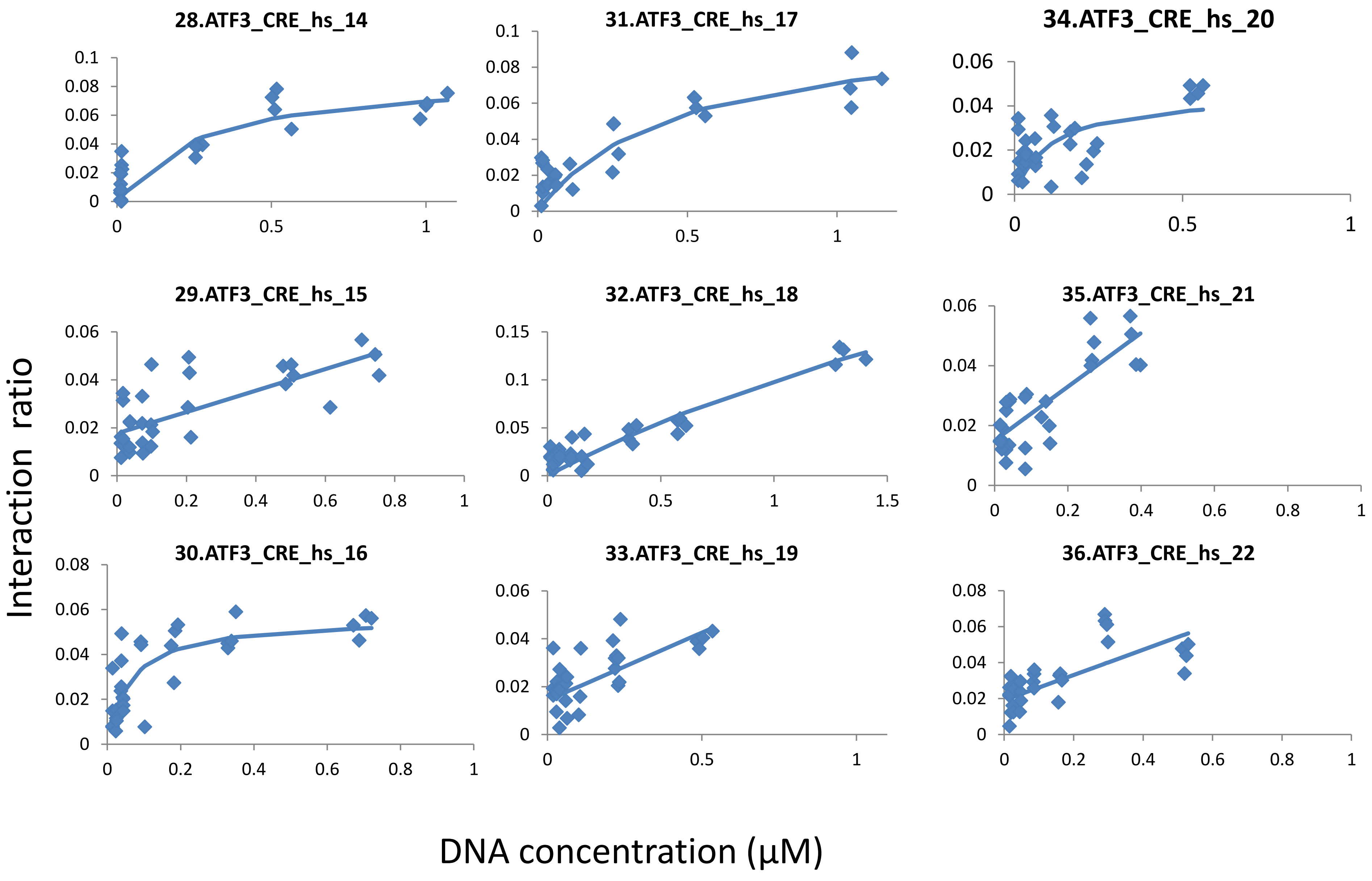
28.ATF3_CRE_hs_14
31.ATF3_CRE_hs_17
34.ATF3_CRE_hs_20
29.ATF3_CRE_hs_15
32.ATF3_CRE_hs_18
35.ATF3_CRE_hs_21
30.ATF3_CRE_hs_16
33.ATF3_CRE_hs_19
36.ATF3_CRE_hs_22
Interaction ratio
DNA concentration (μM)

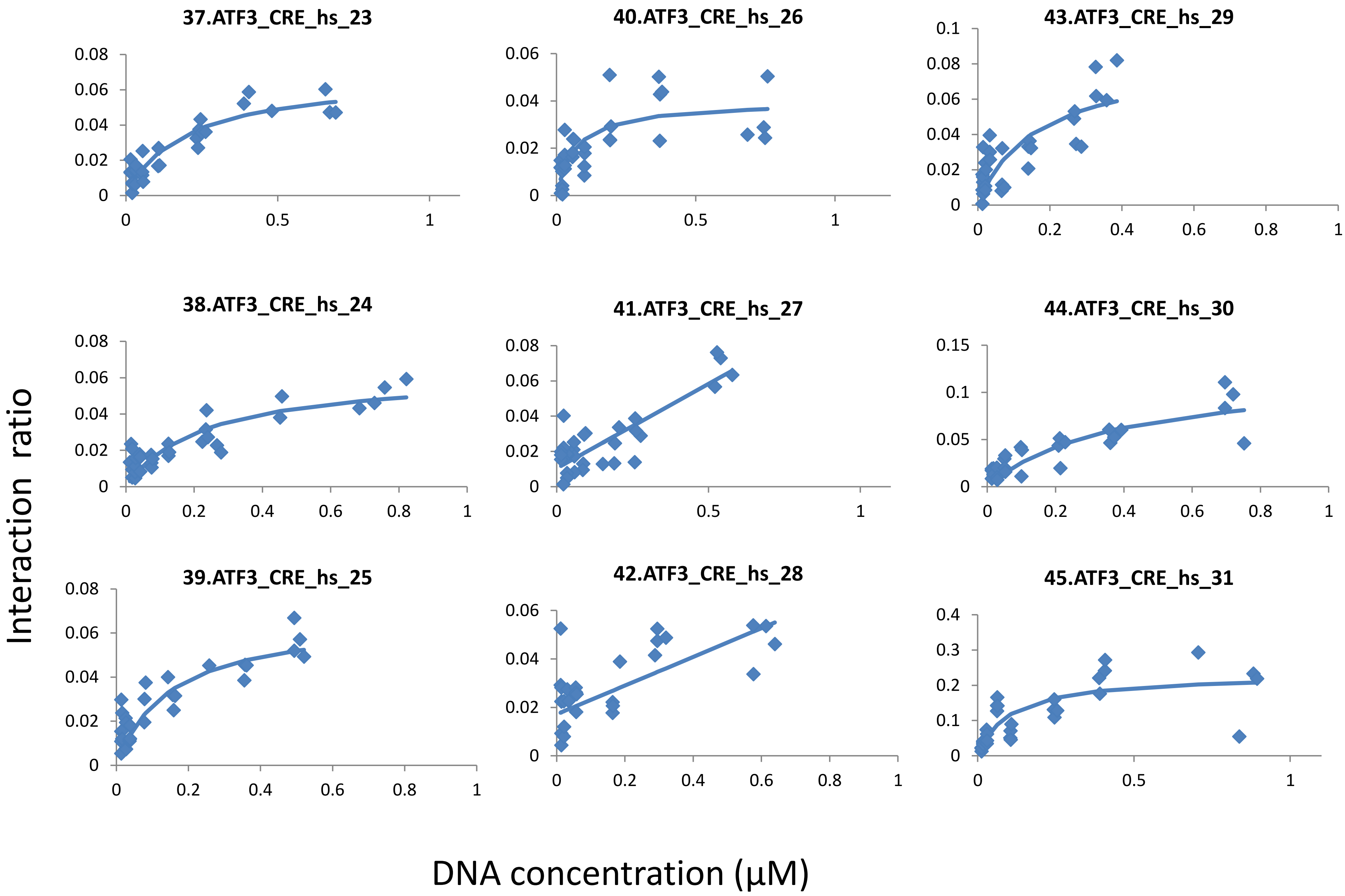

37.ATF3_CRE_hs_23
40.ATF3_CRE_hs_26
43.ATF3_CRE_hs_29
38.ATF3_CRE_hs_24
41.ATF3_CRE_hs_27
44.ATF3_CRE_hs_30
39.ATF3_CRE_hs_25
42.ATF3_CRE_hs_28
45.ATF3_CRE_hs_31
Interaction ratio
DNA concentration (µM)

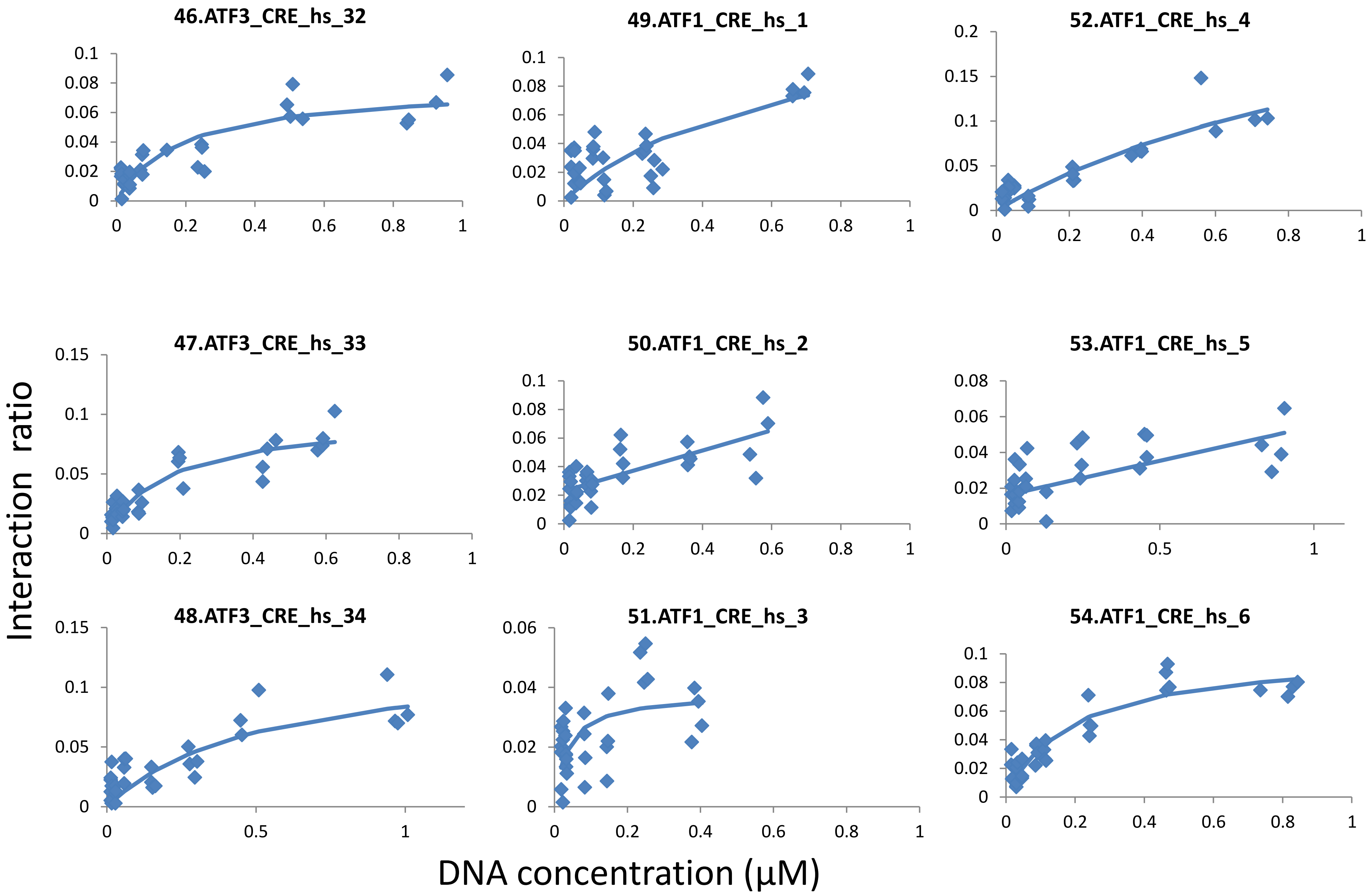
46.ATF3_CRE_hs_32
49.ATF1_CRE_hs_1
52.ATF1_CRE_hs_4
47.ATF3_CRE_hs_33
50.ATF1_CRE_hs_2
53.ATF1_CRE_hs_5
48.ATF3_CRE_hs_34
51.ATF1_CRE_hs_3
54.ATF1_CRE_hs_6
Interaction ratio
DNA concentration (μM)

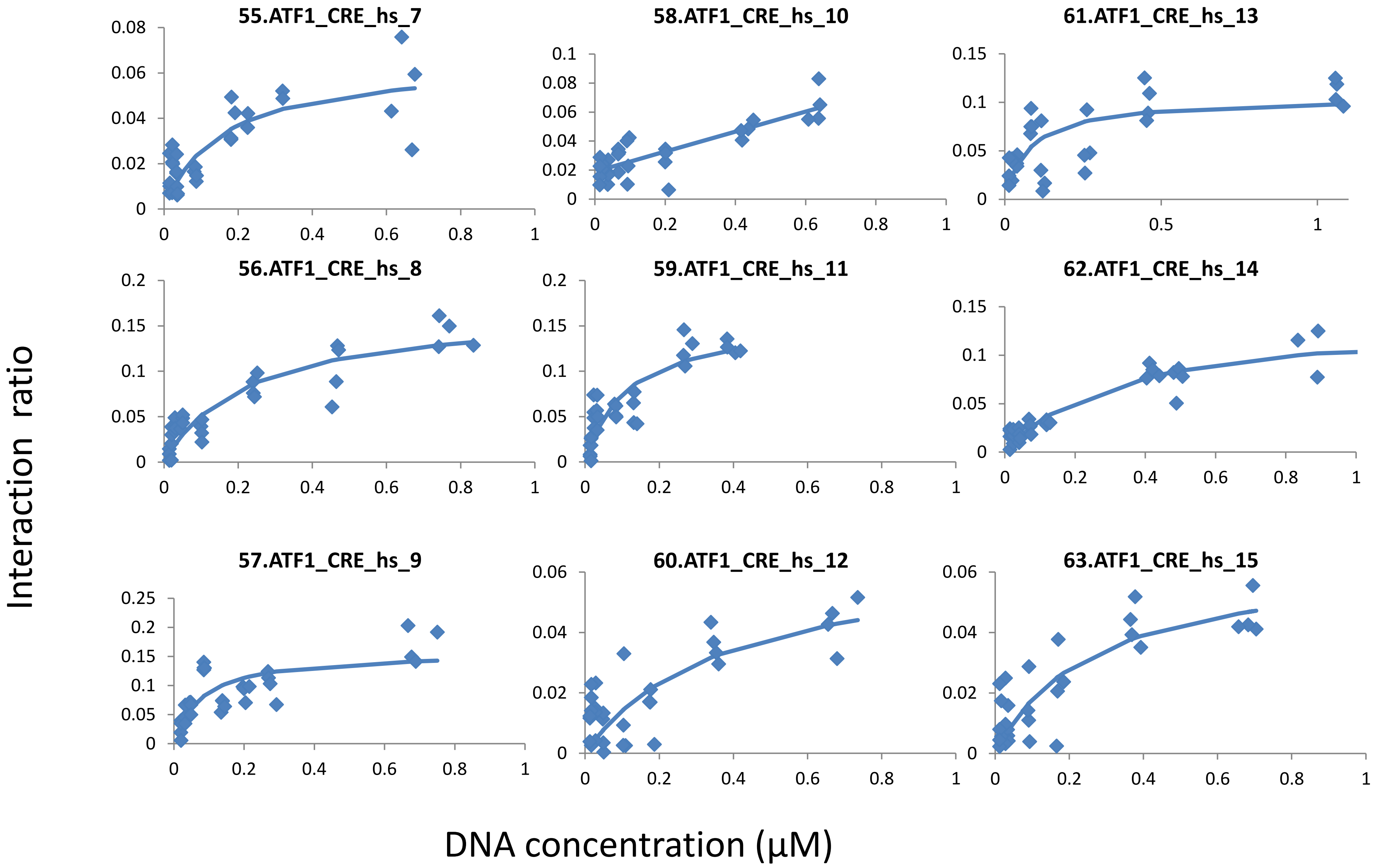
55.ATF1_CRE_hs_7
58.ATF1_CRE_hs_10
61.ATF1_CRE_hs_13
56.ATF1_CRE_hs_8
59.ATF1_CRE_hs_11
62.ATF1_CRE_hs_14
57.ATF1_CRE_hs_9
60.ATF1_CRE_hs_12
63.ATF1_CRE_hs_15
Interaction ratio
DNA concentration (μM)

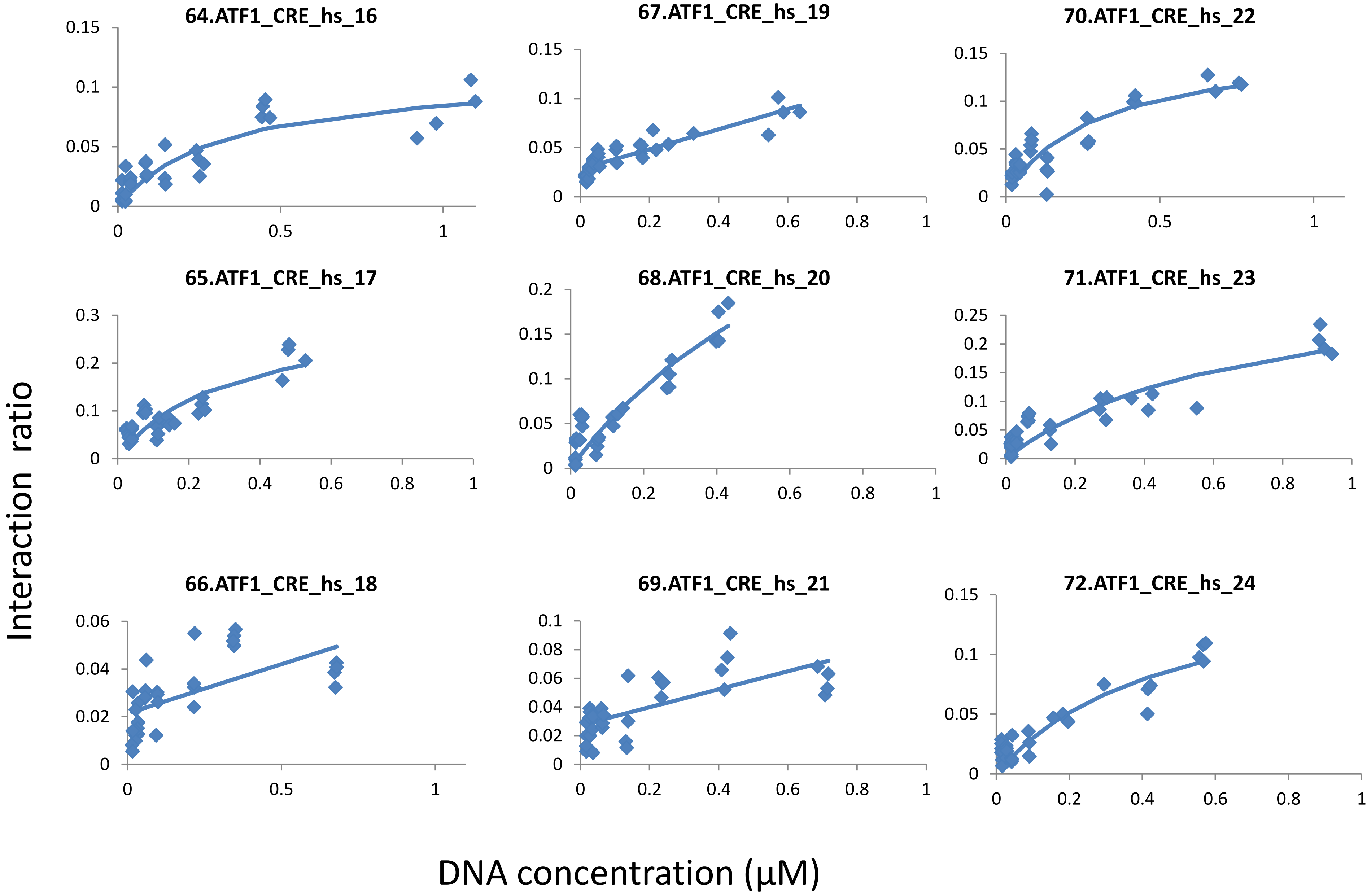
64.ATF1_CRE_hs_16
67.ATF1_CRE_hs_19
70.ATF1_CRE_hs_22
65.ATF1_CRE_hs_17
68.ATF1_CRE_hs_20
71.ATF1_CRE_hs_23
66.ATF1_CRE_hs_18
69.ATF1_CRE_hs_21
72.ATF1_CRE_hs_24
Interaction ratio
DNA concentration (μM)

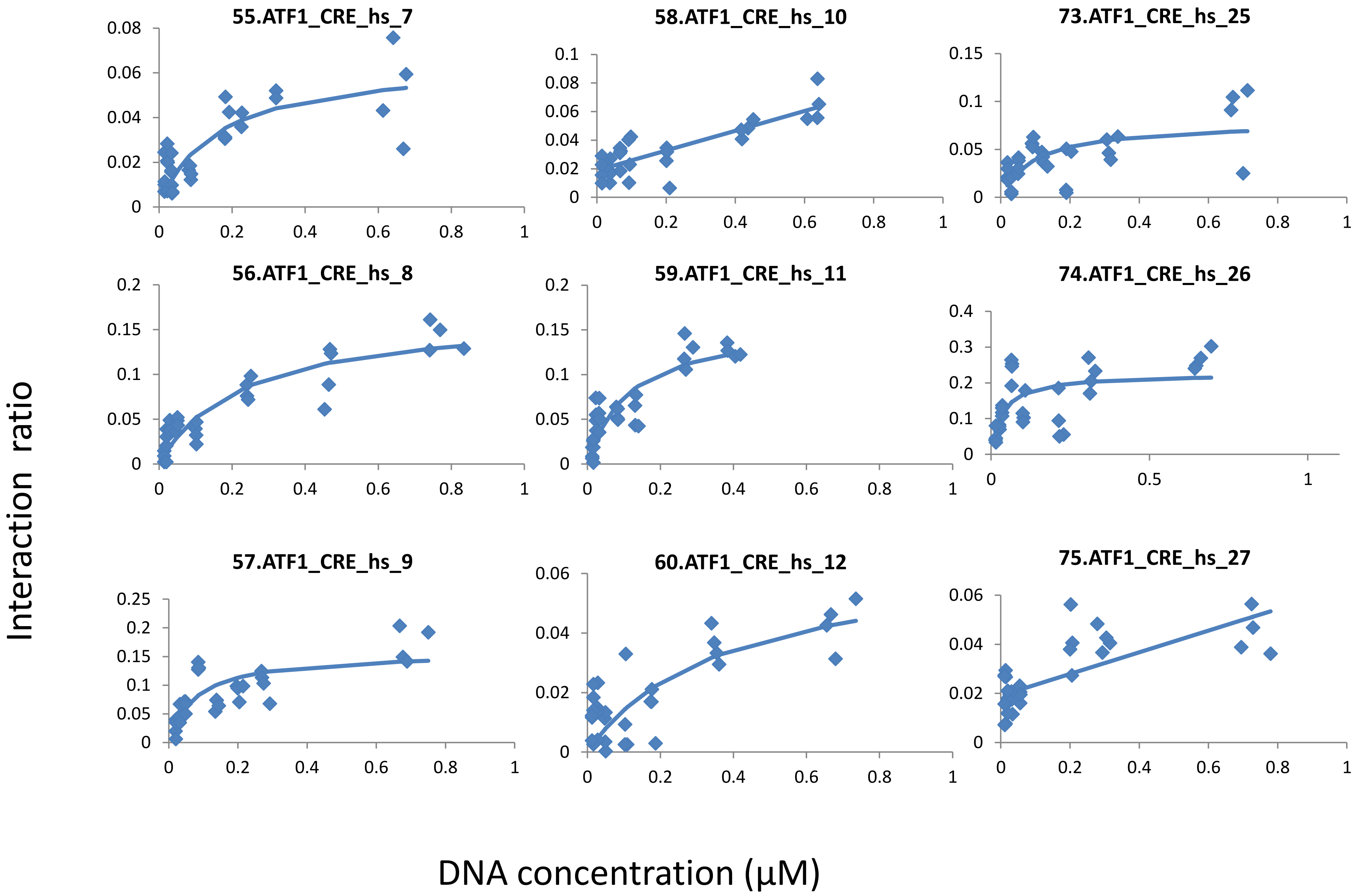
55.ATF1_CRE_hs_7
58.ATF1_CRE_hs_10
73.ATF1_CRE_hs_25
56.ATF1_CRE_hs_8
59.ATF1_CRE_hs_11
74.ATF1_CRE_hs_26
57.ATF1_CRE_hs_9
60.ATF1_CRE_hs_12
75.ATF1_CRE_hs_27
Interaction ratio
DNA concentration (μM)

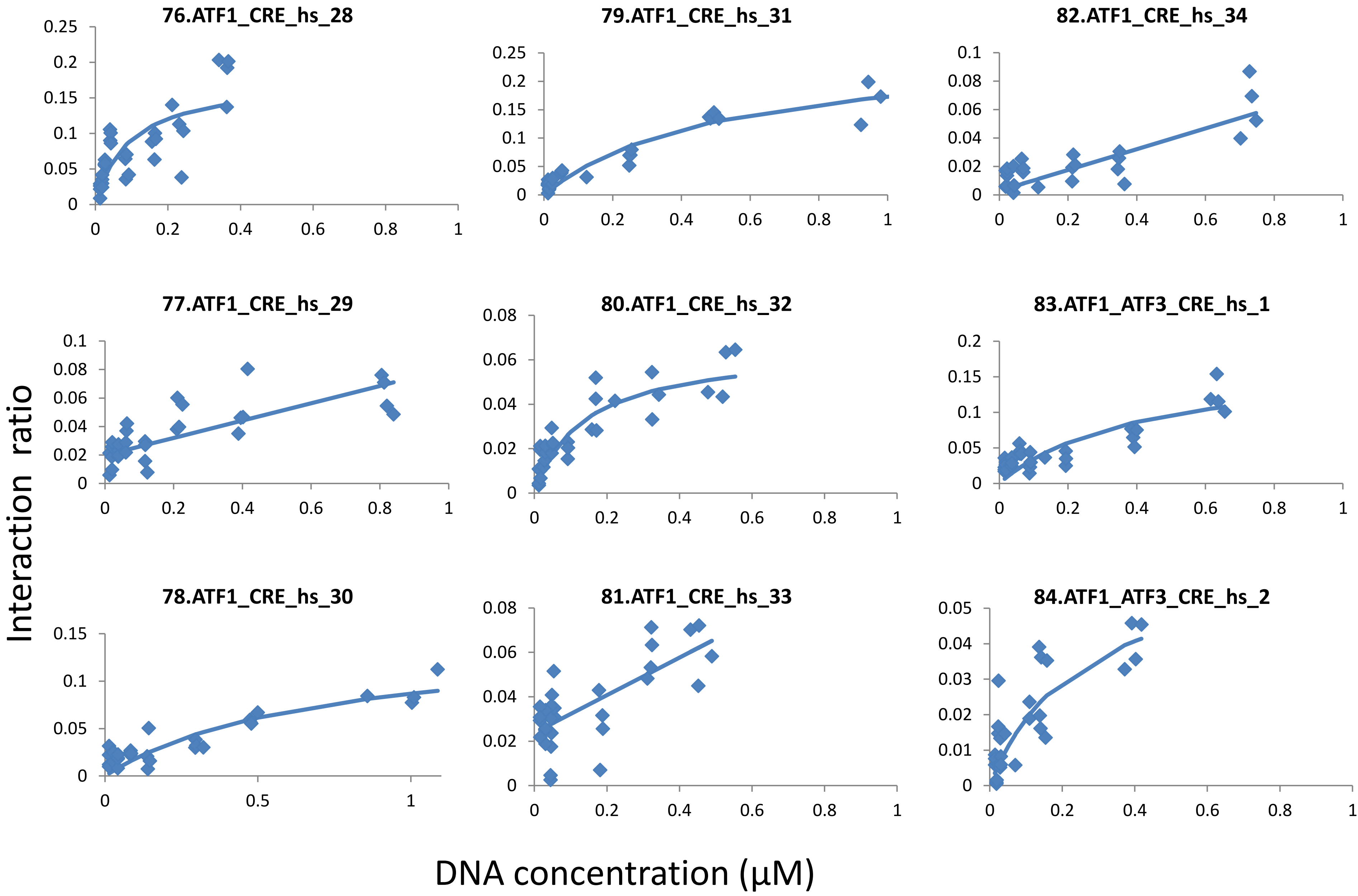

76.ATF1_CRE_hs_28
79.ATF1_CRE_hs_31
82.ATF1_CRE_hs_34
77.ATF1_CRE_hs_29
80.ATF1_CRE_hs_32
83.ATF1_ATF3_CRE_hs_1
78.ATF1_CRE_hs_30
81.ATF1_CRE_hs_33
84.ATF1_ATF3_CRE_hs_2
Interaction ratio
DNA concentration (μM)

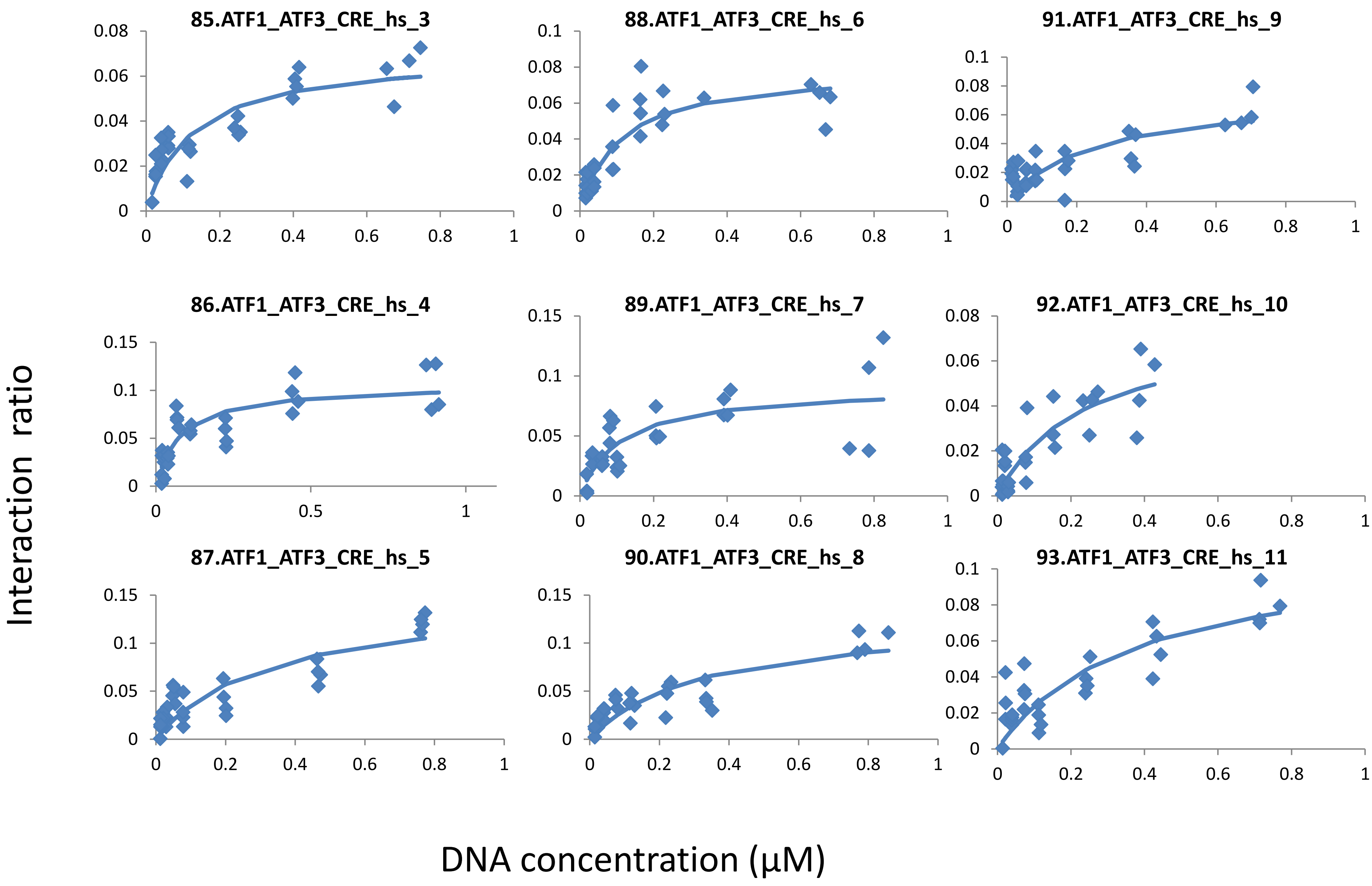

85.ATF1_ATF3_CRE_hs_3
88.ATF1_ATF3_CRE_hs_6
91.ATF1_ATF3_CRE_hs_9
86.ATF1_ATF3_CRE_hs_4
89.ATF1_ATF3_CRE_hs_7
92.ATF1_ATF3_CRE_hs_10
87.ATF1_ATF3_CRE_hs_5
90.ATF1_ATF3_CRE_hs_8
93.ATF1_ATF3_CRE_hs_11
Interaction ratio
DNA concentration (μM)

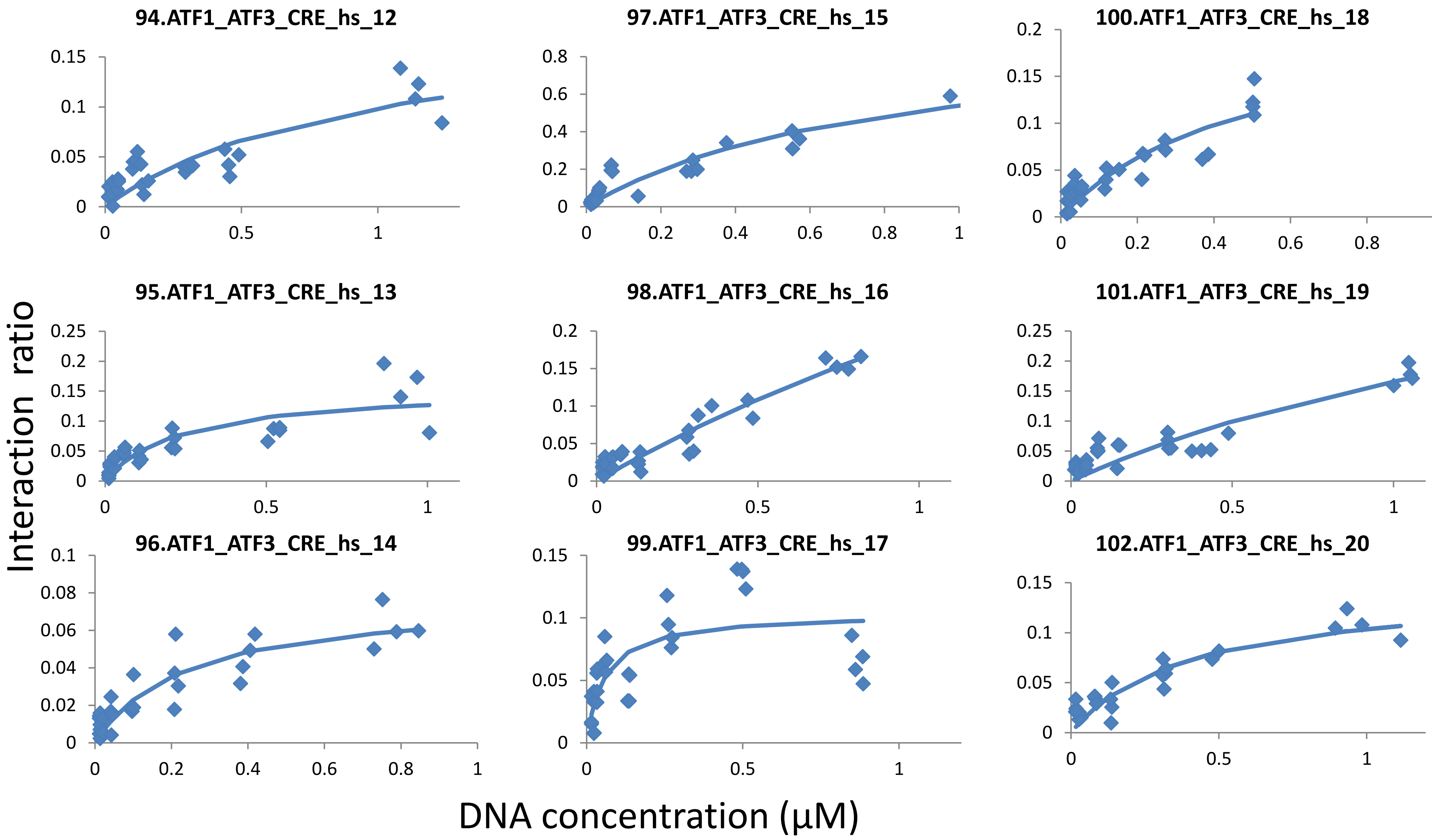
94.ATF1_ATF3_CRE_hs_12
97.ATF1_ATF3_CRE_hs_15
100.ATF1_ATF3_CRE_hs_18
95.ATF1_ATF3_CRE_hs_13
98.ATF1_ATF3_CRE_hs_16
101.ATF1_ATF3_CRE_hs_19
96.ATF1_ATF3_CRE_hs_14
99.ATF1_ATF3_CRE_hs_17
102.ATF1_ATF3_CRE_hs_20
Interaction ratio
DNA concentration (μM)

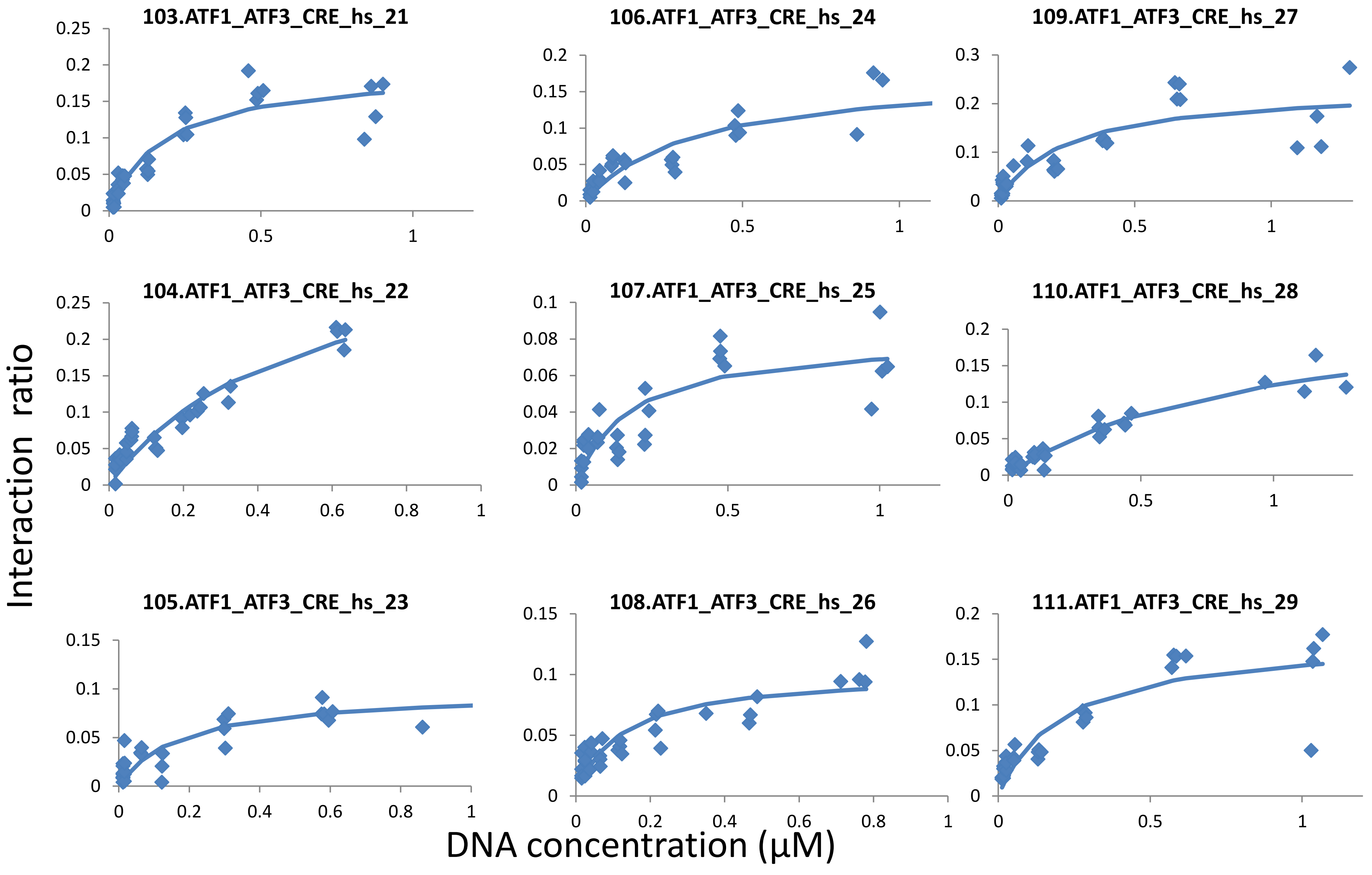

103.ATF1_ATF3_CRE_hs_21
106.ATF1_ATF3_CRE_hs_24
109.ATF1_ATF3_CRE_hs_27
104.ATF1_ATF3_CRE_hs_22
107.ATF1_ATF3_CRE_hs_25
110.ATF1_ATF3_CRE_hs_28
105.ATF1_ATF3_CRE_hs_23
108.ATF1_ATF3_CRE_hs_26
111.ATF1_ATF3_CRE_hs_29
Interaction ratio
DNA concentration (μM)

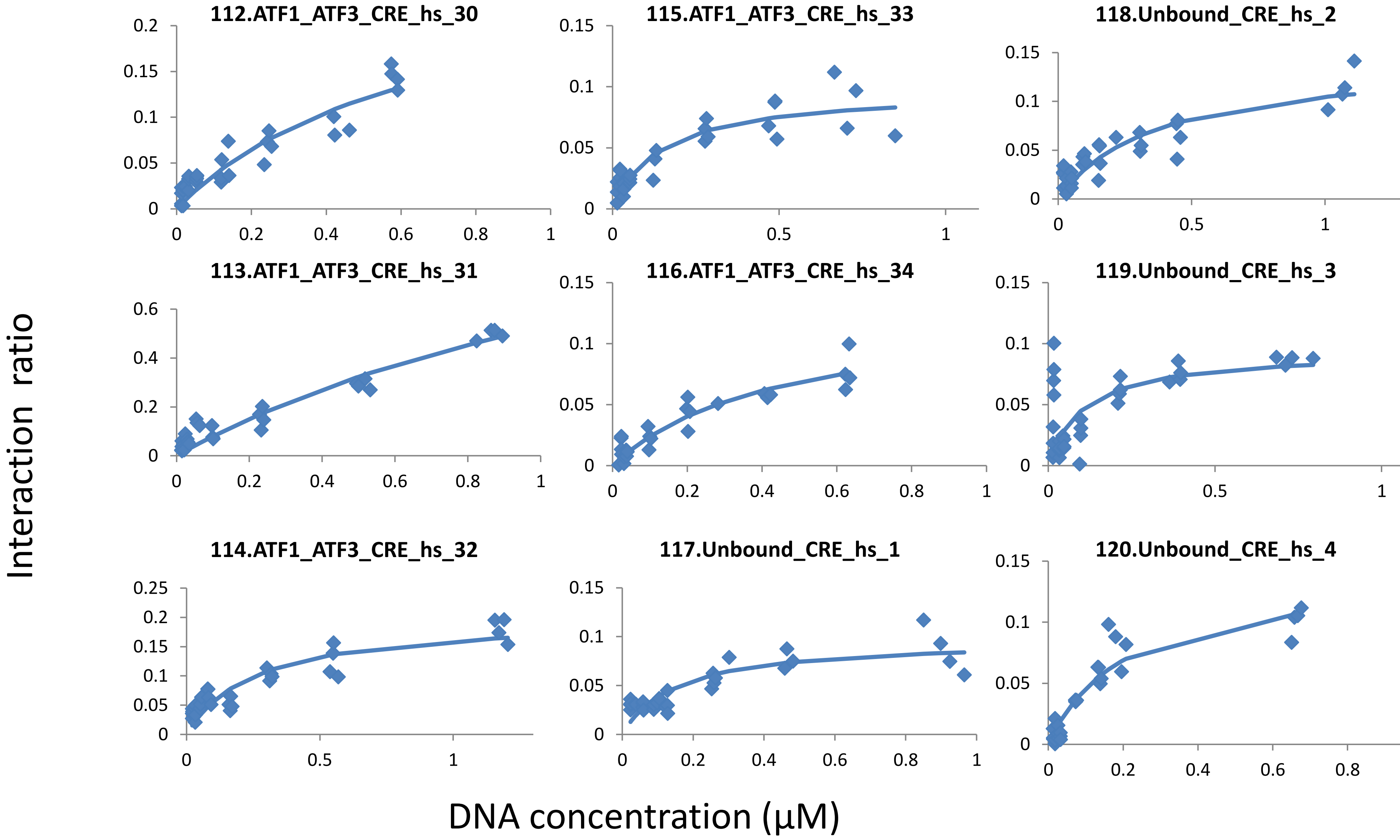
112.ATF1_ATF3_CRE_hs_30
115.ATF1_ATF3_CRE_hs_33
118.Unbound_CRE_hs_2
113.ATF1_ATF3_CRE_hs_31
116.ATF1_ATF3_CRE_hs_34
119.Unbound_CRE_hs_3
114.ATF1_ATF3_CRE_hs_32
117.Unbound_CRE_hs_1
120.Unbound_CRE_hs_4
Interaction ratio
DNA concentration (μM)

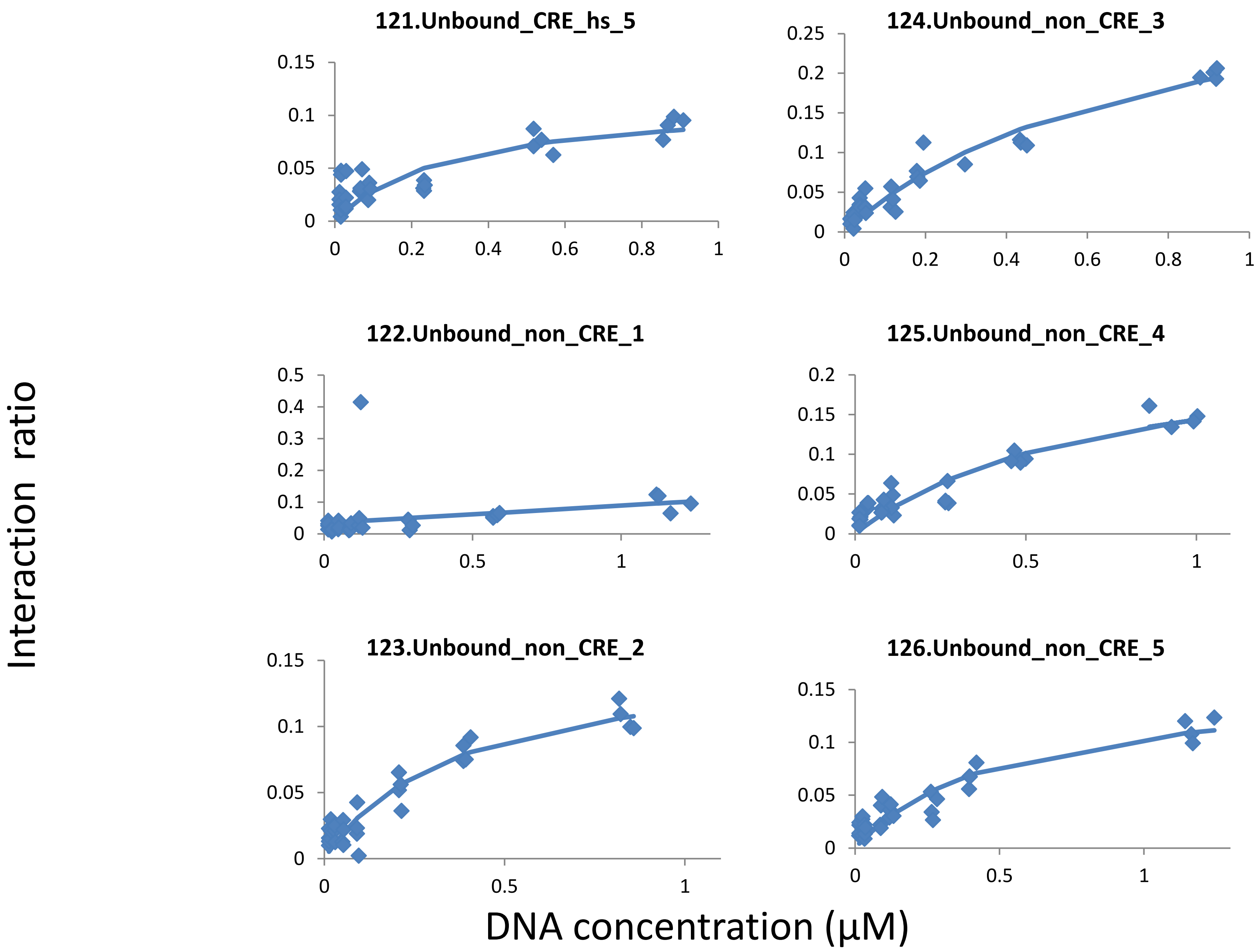

121.Unbound_CRE_hs_5
124.Unbound_non_CRE_3
122.Unbound_non_CRE_1
125.Unbound_non_CRE_4
123.Unbound_non_CRE_2
126.Unbound_non_CRE_5
Interaction ratio
DNA concentration (μM)

Fig. S5

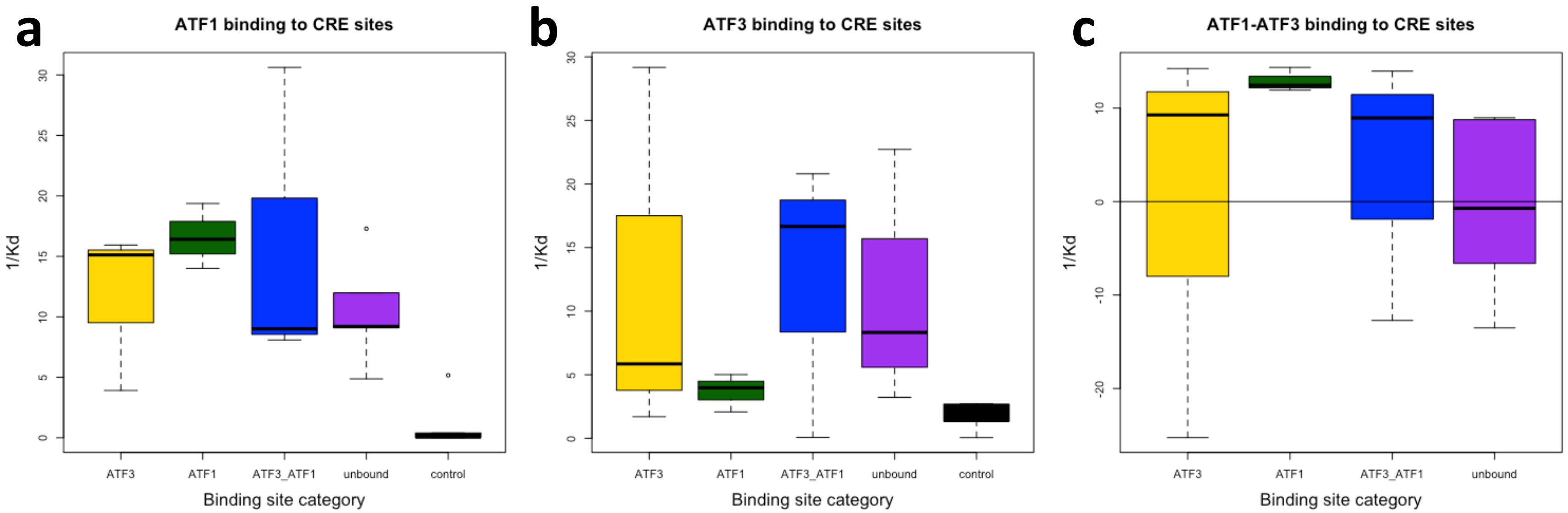


**Figure S5. QPID measurements explain in vivo binding differences between ATF1 and ATF3 to CRE binding sites**. **(a&b)** Boxplots of affinity constants (1/Kd) in different BS categories. ATF1 and ATF3 have higher affinities to sites they bind uniquely than to sites bound by the other protein, unbound and controls. **(c)** Boxplots of affinity constant (1/Kd) differences between Atf1 and Atf3 in different BS categories. ATF1 has higher affinity in sites it binds uniquely than sites bound by ATF3.

# Table. S1

PBM file: ATF1_v1_deBruijn.txt
1647 binding sites

| | oligo 5 | oligo 6 |
|---|---|---|
| HelT | 1.691 | 1.733 |
| MGW | 0.384 | 0.296 |
| ProT | 2.967 | 2.567 |
| Roll | 4.723 | 3.333 |

PBM file: ATF1_v2_deBruijn.txt
1619 binding sites

| | oligo 5 | oligo 6 |
|---|---|---|
| HelT | 1.688 | 1.724 |
| MGW | 0.380 | 0.297 |
| ProT | 3.010 | 2.604 |
| Roll | 4.728 | 3.336 |

ChIP-seq file:
wgEncodeAwgTfbsSydhK562Atf106325UniPk
4310 binding sites

| | oligo 5 | oligo 6 |
|---|---|---|
| HelT | 1.516 | 1.375 |
| MGW | 0.472 | 0.284 |
| ProT | 3.704 | 2.107 |
| Roll | 2.723 | 2.225 |

ChIP-seq file:
K562_ATF1_narrowPeak.bed.txt
7545 binding sits

| | oligo 5 | oligo 6 |
|---|---|---|
| HelT | 1.502 | 1.381 |
| MGW | 0.465 | 0.295 |
| ProT | 3.586 | 2.243 |
| Roll | 2.728 | 2.235 |

**Table S1 - Normalized Euclidean distance of oligo 5 and 6 to average shape features of PBM and ChIP-seq experiments.** Local DNA shape features were predicted for oligo 5 and 6. Average local DNA shape features were predicted for PBM and ChIP-seq binding sites. The Euclidean distances were calculated between the shape features vectors of oligo 5 and 6 and the averages. Each distance was normalized by the square root of the length of the vector.
